# Diminished variability of alpha and beta band-limited power as a neural signature in schizophrenia

**DOI:** 10.1038/s41398-026-04055-w

**Published:** 2026-05-02

**Authors:** Frigyes Samuel Racz, Kinga Farkas, Melinda Becske, Hajnalka Molnar, Zsuzsanna Fodor, Peter Mukli, Gabor Csukly

**Affiliations:** 1 Department of Neurology, The University of Texas at Austin, 1601 Trinity St., Austin, 78712 TX USA; 2 Mulva Clinic for the Neurosciences, The University of Texas at Austin, 1601 Trinity St., Austin, 78712 TX USA; 3 Department of Physiology, Semmelweis University, 37-47 Tuzolto St., Budapest, 1094 Hungary; 4 Department of Psychiatry and Psychotherapy, Semmelweis University, 6 Balassa St., Budapest, 1083 Hungary; 5 Oklahoma Center for Geroscience and Healthy Brain Aging, University of Oklahoma Health Sciences Center, 112 NE 13th St., Oklahoma City, 73117 OK USA; 6 Department of Neurosurgery, University of Oklahoma Health Sciences Center, 1000 N Lincoln Blvd., Oklahoma City, 73104 OK USA; 7 Institute of Preventive Medicine and Public Health, Semmelweis University, 4 Nagyvarad Sq., Budapest, 1089 Hungary

**Keywords:** Schizophrenia, Neuroscience

## Abstract

Spectral features of the electroencephalogram (EEG) are essential for providing clinically relevant biomarkers in schizophrenia (SZ). Despite literature indicating altered short-scale neural dynamics in SZ, however, band-limited power (BLP) indices are rarely assessed in a time-resolved manner. To address this, here we evaluated static and dynamic BLP indices in a sample of 30 SZ patients and 31 healthy control (HC) individuals. Guided by recent findings on power spectral dynamics in SZ, we estimated total, and decomposed fractal and oscillatory BLP in a sliding window manner from resting-state EEG recordings collected in eyes-closed resting-state. The SZ cohort was characterized by elevated baseline of total fractal power (*p* = 0.0015, |*r*| = 0.4073), while its temporal variability was comparable between the two study groups. On the other hand, spectral power in the alpha (*p* < 10^−4^, *d* = 1.0503) and beta (*p* = 0.0022, |*r*| = 0.3925) regimes exhibited reduced fluctuation in SZ compared to HC, with no between-group differences in their baselines. Furthermore, alpha variability could be attributed to alterations in isolated oscillatory activity, while variability in beta-BLP over the dorsal attention network was found correlated with negative symptoms in SZ (Spearman *r* = −0.4994, *p* = 0.0055). Finally, surrogate data testing indicated altered phase dynamics in SZ as a potential mechanism for diminished BLP fluctuations.

## Introduction

Developing and understanding biomarkers of schizophrenia (SZ) is a critical endeavor: such physiology-based features could provide knowledge on potential diagnostic features, future pharmaceutical targets or early indicators of disease progression and therapy response [1–3]. Functional neuroimaging markers – especially those obtained via electroencephalography (EEG) [4, 5] – appear as an intuitive choice for this purpose [6], with resting-state EEG providing a fast and convenient way to infer on brain dynamics. Currently, the most consistent resting-state EEG alterations identified in SZ include an increase in delta- and theta-band activity, while a decrease in alpha power [7, 8], though contradictory findings are also reported [9]. Furthermore, extensive dynamic functional connectivity literature indicates that short-scale neural dynamics are altered in SZ, often in a way that is undetectable for conventional, time-invariant analytical approaches [10, 11]. Interestingly, however, despite all this evidence, the temporal dynamics of resting-state EEG power spectral features are rarely considered in SZ.

Altered cortical excitation/inhibition (E/I) ratio is a pathological feature that is considered central in SZ [12] with explicit potential for clinical utility [13, 14]. The 1/*f* slope ($\beta$) of electrophysiological signals is hypothesized to reflect on the E/I ratio [15], and adequate estimation of $\beta$ requires the separation of oscillatory from broadband fractal components in the power spectrum [16, 17]. Regarding SZ, a recent systematic review [18] concluded – from a sample of 8 papers – that $\beta$ does not reveal a consistent disease-characteristic pattern and thus more research is warranted; however, while methodologies differed, none of these articles considered the temporal evolution of $\beta$. On the other hand, separating fractal and oscillatory components of the power spectrum is not only relevant for obtaining unbiased estimates [19]; previous research also indicates that the two components likely reflect on distinct neuronal processes and corresponding behavioral aspects [20–22]. Furthermore, it is hypothesized that aberrant phase dynamics resulting in temporal imprecision are a core pathological feature of SZ, which can simultaneously explain inefficiencies in processing sensory stimuli [23, 24] and provide novel diagnostic biomarkers [25, 26].

Along these considerations and building on previous research [27], we recently showed [28] that while the overall baseline (average over time) of $\beta$ is comparable in SZ and healthy controls (HC), the patient group showed significantly diminished temporal variability in $\beta$ in the 20–45 Hz regime. That work, however, focused exclusively on the spectral slope – omitting conventional EEG measures such as spectral power in canonical frequency regimes [7, 9] –, as well as potential explanatory mechanism were not explored. Therefore, our goal here was to provide a comprehensive picture and understanding of dynamic EEG alterations in SZ by conducting a time-resolved analysis of EEG spectra decomposed into aperiodic $1/f$ and oscillatory components. We expected reduced temporal variability of both fractal and oscillatory spectral power in SZ, which correlates with clinical symptom scores as captured via the Positive and Negative Syndrome Scale (PANSS) [29], and hypothesized that these alterations originated from aberrant phase dynamics.

## Materials and methods

### Participants and clinical measures

We analyzed EEG recordings of the same study cohorts as in our recent study [28]. The SZ group was comprised of 30 SZ patients (11 female, age: 33.07 ± 9.73 years), with an age- and sex-matched healthy control (HC) group of 31 individuals (13 female, age: 33.06 ± 10.31 years). There was no statistically significant difference between the two groups in terms of age, proportion of sexes, or years in education (Table 1). Schizophrenia patients were ON medication at the time of recording. For further study cohort details on inclusion/exclusion criteria and medication, please see Supplementary Material. The Semmelweis Regional and Institutional Committee of Science and Research Ethics reviewed and approved the study (approval number: 197/2015), which was conducted in line with the Declaration of Helsinki. All participants were informed about study details and provided prior written consent for participation.

**Table 1:** Demographic information of study groups.

Variable	SZ (*n* = 30)	HC (*n* = 31)	Statistics	*p*-value
**Age**	33.07 ± 9.73	33.07 ± 10.48	t = 0.0	*p* = 1.0
**Sex**	11F/19M	13F/18M	${\chi }^{2}$ = 0.2278	*p* = 0.5982
**Years in education**	16 [8; 18]	16.5 [12; 18]	z = 1.1085	*p* = 0.2676
**Illness duration**	8.17 ± 8.57			
**CPZ equivalent dose**	394.16 ± 346.01			

*SZ* schizophrenia, *HC* healthy control, *CPZ* chlorpromazine equivalent dose.

### EEG recording, quality control and pre-processing

**Fig. 1:**
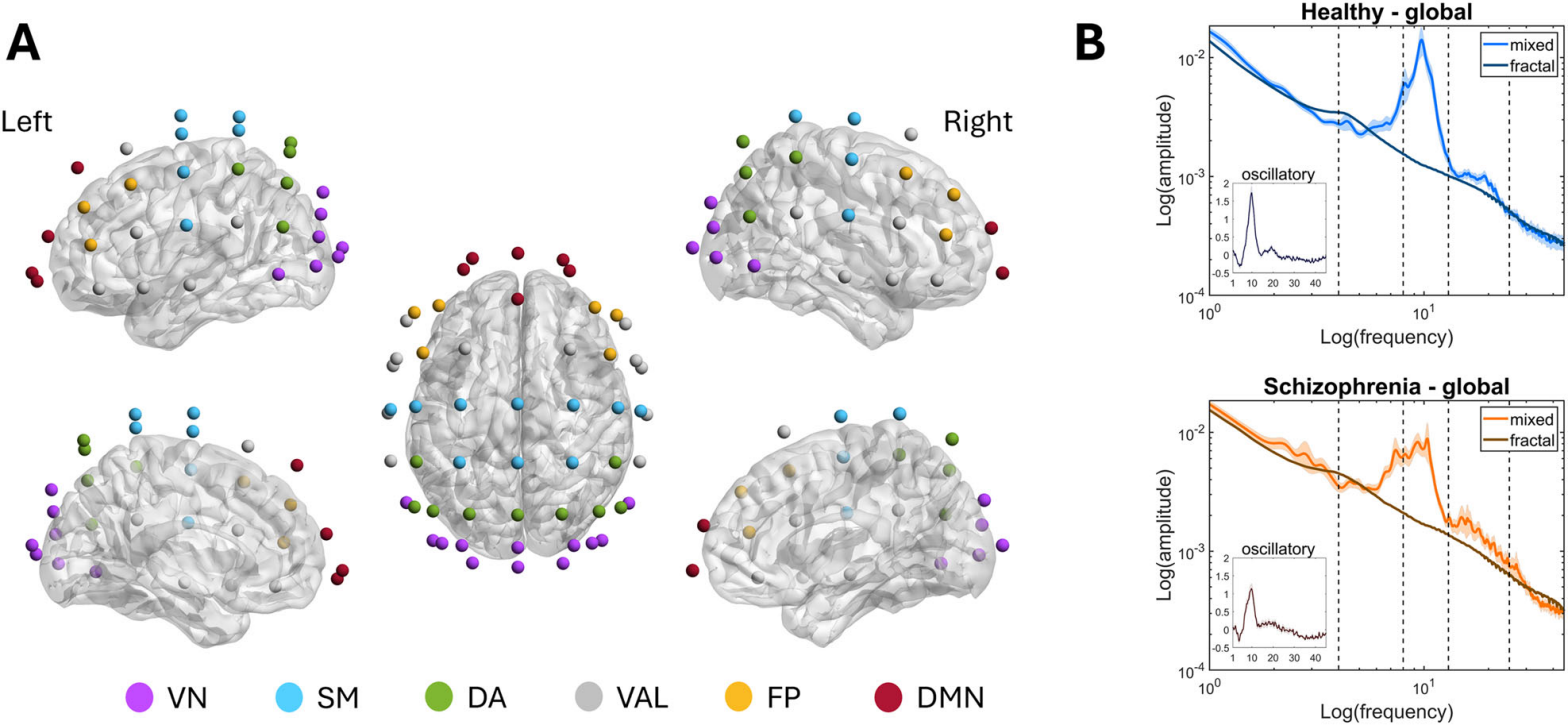
Electrode montage, channel groupings and illustration of the IRASA method. Panel **A** shows the electrode layout, where color indicates the association of each electrode to one of the six resting-state networks. Panel **B** illustrates the separation of the fractal and oscillatory (inset plots) components from the raw (mixed) power spectrum for healthy (upper) and schizophrenia (lower). In both cases, the group average of global spectra (taken as the average of all channels) obtained from eyes-closed condition is presented for illustration. Shaded areas indicate the standard error of the mean, and dashed lines denote the boundary frequencies between the five investigated frequency bands (delta: 1–4 Hz; theta: 4–8 Hz; alpha: 8–13 Hz; beta: 13–25 Hz; gamma: 25–45 Hz). IRASA: irregular-resampling auto-spectral analysis; VN: visual network; SM: somatomotor network; DA: dorsal attention network; VAL: combined ventral attention and limbic networks; FP: frontoparietal network; DMN: default mode network.

EEG was collected in a single session from 64 standard 10–10 locations at 1000 Hz resolution using a Neuroscan amplifier. Channels were referenced to linked mastoids and electrode impedances were kept under $5k\Omega$. Resting state data was collected for two minutes in eyes closed (EC) resting-state condition. Signal artifacts and transitory noise components can severely bias estimates from time-resolved spectral analysis; to counter this, we employed a strict procedure to select clean, continuous EEG segments for analysis. In that, data was visually inspected by two investigators separately, blinded to study group association. Both investigators first labeled data segments in the complete dataset as clean (i.e., free of overt artifacts) and noisy. Then, the quality assessments were synthesized, and data segments were identified for each subject which were deemed admissible by both investigators independently. As a result of this screening procedure, channels AF7, AF8, AFz, F1, F2 TP7 and TP8 (along with M1/M2) were excluded from further analysis as they were identified as noisy/bad in more than 10 participants according to standard criteria [28], resulting in a final channel count of 55. Furthermore, once admissible segments were collected from the complete dataset, 30 s was identified as the longest continuous duration that was available for most participants. In line, 8–8 participants were excluded from the original cohort of 39 HC and 38 SZ individuals, where 30 s of admissible data was not available, resulting in a final sample of 31 and 30 HC and SZ participants, respectively. For each participant, the middle 30 s of admissible data was retained for analysis. Exact time stamps for included data are reported in the **Online Supplement** (see **Data Availability Statement**). The electrode montage (generated using the BrainNet Viewer tool [30]) is illustrated on Fig. 1A.

Data was processed and analyzed in Matlab (MathWorks, Natick, MA) using the EELAB toolbox [31] along with custom functions and scripts. Data was first band-pass filtered between 0.1 and 128 Hz using the default finite impulse response filter of EEGLAB with additional notch filters at 50 Hz and 100 Hz, and then downsampled to 256 Hz for computational efficiency. EEG signals were then decomposed via independent component analysis (ICA) and artifacts corresponding to non-neural sources (such as potential eye movements, muscle activity, heart activity, head movement) were identified and removed in an automatized manner using the Multiple Artifact Rejection Algorithm (MARA) toolbox [32, 33] before reverse-ICA transformation. This resulted in the removal of 27.44 ± 8.22 out of 55 components, respectively. There was no statistical difference (*p* > 0.35) in the number of removed components between HC and SZ (see **online** Supplementary Material). Finally, after re-referencing to the common average, EEG signals were standardized (z-scored) according to $z(t)=(x(t)-\mu (x))/\sigma (x)$, where $x(t)$ is the original time series with $z(t)$ its z-scored version, and $\mu (x)$ and $\sigma (x)$ are the mean and standard deviation of $x(t)$, respectively. As z-scoring renders all time series zero-centered and with a standard deviation of 1, this step was introduced to help avoid potential confounding effects on power estimates by varying impedances across channels and subjects.

### Estimating spectral power

We employed a sliding window approach to obtain time-resolved band-limited power (BLP) estimates after separating $1/f$ aperiodic and oscillatory components of the EEG spectra. In that, we utilized a window size of 8 s and a step size of 1 s (87.5% overlap), resulting in 23 estimates from the 30-second epochs. Note that with z-scoring performed on the complete 30-second segments, these window estimates reflect distribution of power along the complete broadband spectrum, while at the same time capturing relevant dynamics over the 30-second period (in contrast to z-scoring in every window individually). Similar to our previous approach [28], in every window we decomposed the raw (mixed) power spectrum into fractal and oscillatory components using the Irregular Resampling Auto-Spectral Analysis (IRASA) technique [16]. The IRASA technique is illustrated on Fig. 1B, while for details, please see the original publication of Wen & Liu [16]. Frequency resolution was set to 0.0625 Hz and the resampling factor $h$ ranged from 1.1 to 2.6 in increments of 0.1 [28]. With these parameter settings the frequency regime over 45 Hz was inaccessible due to the filtering effects introduced by resampling in IRASA [34]. For mixed (${mixd}$) and fractal (${frac}$) spectra power estimates were log-transformed, while for oscillatory (${osci}$) spectra – where positive power is not strictly ensured by IRASA – we computed the difference between log-transformed mixed- and log-transformed fractal-power (i.e., the proportion of oscillatory power). Ultimately, this analysis produced three spectrograms (${mixd}$, ${frac}$ and ${osci}$) of size $1024\times 23$ (frequencies × time windows).

### Frequency-wise analysis

For an overall insight on brain dynamics in HC and SZ, we evaluated global spectral patterns on the level of frequencies. For this analysis, the mean (baseline) and standard deviation (fluctuation) over time windows were computed from the log-transformed ${mixd}$, ${frac}$ and ${osci}$ spectrograms, and the global average was taken over all channels. Then, all outcomes were collapsed over frequency bins of 2 Hz to reduce dimensionality, resulting in 22 bin estimates for all $\mu$ and $\sigma$ measures in the 1–45 Hz regime. We denote the mean and standard deviation of spectral power ($P(f)$) with $\mu (\cdot )$ and $\sigma (\cdot )$, respectively, with the spectrum type indicated in subscript e.g., the standard deviation of fractal power is $\sigma ({P}_{{frac}}\left(f\right))$.

### Band-limited power analysis

Besides frequency-level analysis, the following conventional frequency bands were defined: delta ($\delta$): 1–4 Hz, theta ($\theta$): 4–8 Hz, alpha ($\alpha$): 8–13 Hz, beta ($\beta$): 13–25 Hz and gamma ($\gamma$): 25–45 Hz. BLP estimates in all regimes were obtained via averaging and characterized by their temporal $\mu$ and $\sigma$, taken over the 23 time windows. Importantly, the fractal signal component represents broadband activity and therefore it should not be characterized over narrow frequency bands. Instead, there are various suitable approaches. Most appropriately, one can characterize the fractal component by its power-law scaling exponent, such as in our previous analysis [28] (also see below). Alternatively, one can compute the total fractal power via integrating the fractal power spectrum over the investigated frequency regime, such as in [22]. As our previous analyses indicated spectral bimodality in separate low- (1–4 Hz) and high-frequency (20–45 Hz) regimes [28], here we take the latter approach. Consequently, we considered the following time-resolved characterization of power spectra: ${mixd}$ and ${osci}$ BLP in five frequency bands, and total fractal power (TFP). For the former indices, we will use the following notation: frequency band is indicated by its preceding Greek-letter symbol (e.g., α), physiological state (EC) is indicated in superscript, spectrum type (${mixd}$ and ${osci}$ for raw/mixed and oscillatory spectra, respectively) is indicated in subscript, and the mean or variance is indicated by encapsulating $\mu (\bullet )$ and $\sigma (\bullet )$, respectively. For example, temporal fluctuation of eyes-closed BLP obtained from oscillatory spectra in the alpha regime is denoted σ($\alpha {{BLP}}_{{osci}}^{{EC}}$).

For dimensionality reduction and simplified physiological interpretability, channel-level estimates were collapsed over six, established resting-state brain networks [35]. We followed our previous approaches in this process [28, 36, 37] – building on the work of Giacometti and colleagues [38] – and assigned EEG channels to the Visual Network (VN), the Somatomotor Network (SM), the Dorsal Attention Network (DA), the Frontoparietal Network (FP), the combined Ventral Attention and Limbic Networks (VAL) and the Default Mode Network (DMN). In addition, we also performed all analyses on the global average indices taken over all 55 channels. The RSN assignment is illustrated on Fig. 1A. Note that the overall aim of this procedure was to reduce dimensionality while preserving broad-sense topological organization at the same time, and not to infer on the activity of specific anatomical structures.

A summary of analysis aspects is provided in Table 2. Note that as part of the IRASA analysis [16], $1/f$ spectral slope estimates in low- (1–4 Hz, ${\beta }_{{lo}}$) and high-frequency (20–45 Hz, ${\beta }_{{hi}}$) regimes were also obtained as in [28], both on the level of channels and RSNs.

**Table 2:** Summary of analysis aspects and their short description.

Aspect	Notation	Summary
Spectral power	BLP	Band-limited power (BLP) is obtained via Welch’s periodogram method, then integrated in the given frequency range and log-transformed to facilitate normality.
Frequency range	$\delta$, $\theta$, $\alpha$, $\beta$ and $\gamma$	Canonical frequency bands Delta ($\delta$): 1–4 Hz, Theta ($\theta$): 4–8 Hz, Alpha ($\alpha$): 8–13 Hz, Beta ($\beta$): 13–25 Hz and Gamma ($\gamma$): 25–45 Hz.
Spectrum type	${mixd}$ and ${osci}$	Power spectra separated by the IRASA method [16]. The raw (${mixd}$) power spectrum is assumed to be the sum of $1/{f}^{\beta }$ fractal (${frac}$) and narrow-band oscillatory (${osci}$) components, with the latter obtained by subtracting the fractal from the raw spectrum.
Statistical measure	$\mu (\cdot )$ and $\sigma (\cdot )$	EEG is analyzed in a time-resolved, sliding window fashion. Note that continuous 30-second EEG segments are standardized to ensure identical distribution of total power among participants, but individual windows (epochs) are not to allow for BLP dynamics to emerge. Then, brain activity is characterized by the baseline i.e., mean ($\mu (\cdot )$) and extent of temporal fluctuation i.e., standard deviation ($\sigma (\cdot )$) of BLP estimates.
Topology	VN, SM, DA, VAL FP, DMN and Global	EEG channels are collapsed onto brain resting-state networks: the Visual Network (VN), the Somatomotor Network (SM), the Dorsal Attention Network (DA), the Frontoparietal Network (FP), the combined Ventral Attention and Limbic Networks (VAL) and the Default Mode Network (DMN). In addition, global average indices taken over all 55 channels are also assessed. See Fig. 1A for channel assignment.

*EEG* electroencephalography, *IRASA* Irregular Resampling Auto-Spectral Analysis.

### Statistical evaluation

In the frequency-wise analysis, estimates ($\mu$ and $\sigma$ from ${mixd}$, ${frac}$ and ${osci}$ at the frequency-level) were contrasted between HC and SZ the following way. All statistical tests performed were two-sided. Normality of variables was first assessed via Lilliefors test, then a two-sample t test or Mann-Whitney U test was utilized accordingly. Similarity of variances was assessed using F-test or Levene’s test. For each case (e.g., $\sigma$ from ${mixd}$ spectrograms), outcomes were adjusted for multiple comparisons (22 comparisons) using the False Discovery Rate (FDR) method of Benjamini and Hochberg [39]. Furthermore, to assess if the time-variance in ${mixd}$ and ${osci}$ spectra were evenly distributed over the 1–45 Hz frequency regime, we performed within-group analyses in HC and SZ separately, where $\sigma$ estimates from ${mixd}$ and ${osci}$ were contrasted in a bin-to-bin manner along similar principles. This produced $({N}_{f}\times \left({N}_{f}-1\right))/2=231$ comparisons in each case, where ${N}_{f}=22$ is the number of frequency bins, which were adjusted using FDR.

For BLP-level analysis, spectral features were contrasted between the HC and SZ groups on global-to-global and RSN-to-RSN levels. Channel-to-channel comparisons were performed only for exploratory and visualization purposes according to similar principles. In all cases, data normality was first probed with Lilliefors test, then either two-sample t-test or Mann-Whitney U test was employed accordingly. In both channel-wise and RSN-wise analyses, outcomes were adjusted for multiple comparisons using FDR. For the channel-wise analysis this involved 55 channels × (5 frequency bands × 2 spectrum types + TFP) × 2 measures = 1210 variables, while (6 RSNs + ${global}$) × (5 frequency bands × 2 spectrum types + TFP) × 2 = 154 variables for the RSN-wise analysis. Effect sizes were characterized via Cohen’s *d* for normally distributed data, while according to $r=\left|z\right|/\sqrt{N}$ otherwise, where $z$ and $N$ are the standardized test statistic and the total sample size, respectively [40]. Note that throughout the main text we only report details of statistical tests that indicated significant effect; detailed reports of all hypothesis tests are reported in the online supplement in tabular format and as MATLAB workspaces (see **Data Availability Statement**).

Those global and RSN-level spectral features that revealed significant between-group difference – and therefore could be considered characteristic traits of SZ – were further subjected to correlation analysis with general (GEN-PANSS), negative (NEG-PANSS), positive (POS-PANSS) and total (SUM-PANSS) symptom scores. The effect of possible confounding variables age, sex, years in education, disease duration and CPZ equivalent dose was estimated in both spectral indices and PANSS scores via multiple linear regression. Then, normality of the residuals was assessed via Lilliefors test, and the relationship between spectral indices and clinical symptoms were characterized via Pearson or Spearman correlation coefficient accordingly. Outcomes were not adjusted for multiple comparisons and therefore these outcomes should be treated as exploratory. Effect sizes were interpreted according to Cohen’s guidelines, with Pearson or Spearman ${|r|}=0.1$, $0.3$ and $0.5$ indicating small, moderate or large effect [41].

Since patients were ON medication at the time of recording, we also performed correlation analyses between global EEG indices and CPZ equivalent dose values along similar considerations as described previously. Only global EEG indices that showed significant between-group differences in SZ were included in this analysis. While the goal was to assess correlation between EEG markers and CPZ, other confounders such as age, sex, years in education and disease duration were regressed out from both EEG indices and CPZ values. Investigating the relationship between PANSS scores and CPZ was conducted according to the same principles.

Resting alpha BLP is hypothesized to reflect inhibitory activity [42, 43], and thus its changes might be correspondent to changes in the E/I ratio [44]. To this end we investigated how patterns observed in oscillatory alpha BLP resembled those in ${\beta }_{{hi}}$. For this purpose, we took global oscillatory $\alpha {BLP}$ and global ${\beta }_{{hi}}$ estimates obtained from all 23 time windows and utilized linear mixed-effects (LME) modeling with participant included as a random effect to assess how the time evolution of ${\beta }_{{hi}}$ follows that of $\alpha {{BLP}}_{{osci}}^{{EC}}$. This analysis was carried out separately for the HC and SZ groups.

### Surrogate data testing

To test if observed dynamic fluctuations in spectral power are of nonlinear origin, we utilized the phase randomization technique introduced by Theiler and coworkers [45]. In that, we took the original, 30-second (pre-processed) EEG time series, performed Fourier transformation, and then randomized the phases before regaining the signal via inverse Fourier transformation. Therefore, if observed changes in spectral dynamics were a consequence of nonlinearity, we should see reduced temporal variability of BLP in the surrogate time series. For every participant, a set of *n* = 100 surrogates were generated, and we replicated the sliding window analysis on these surrogate datasets. Note that since the IRASA procedure has very high computational demand [22], this analysis was only carried out for mixed – but not isolated oscillatory – BLP estimates. In that, the power spectrum in each window was reconstructed using the Welch periodogram method with Hanning windowing (same as in IRASA). We performed both within- and between-group analyses regarding phase randomized surrogates. In the former, presence of nonlinearity was statistically confirmed for a given measure if a z-test indicated with *p* < 0.05 certainty that the true spectral index (e.g., $\sigma (\alpha {{BLP}}_{{mixd}}^{{EC}})$) was not obtained from the same distribution as those obtained from surrogates. Between-group analyses were also performed on surrogate indices; if between-group differences in true measures disappear in phase-randomized surrogates, it is confirmed that the differences were driven by abnormal phase dynamics [46]. In this analysis, for a given measure the average value over 100 surrogates were taken, and between-group comparisons were performed as described previously.

### Confirmatory analyses

To assess the potential effect of window-size on dynamic power spectral estimates, we repeated our complete analysis pipeline using 4- and 2-second window sizes. To remain consistent in our approach we utilized an overlap of 87.5% between consecutive windows (0.5- and 0.25-second step size). These analyses produced 53 and 113 time windows for window sizes 4- and 2-seconds, respectively. Power spectral and statistical analyses were performed as described before.

One setback of the sliding window approach is that at a fixed window length there is a discrepancy in the representation of various frequency components (i.e., the window contains fewer cycles for low-frequency components, and vice versa). To examine if this had a significant effect on baseline and dynamic power spectral estimates, we devised a different approach, where instead of utilizing a fixed window length, spectral power at each frequency was estimated from windows containing an equal number of cycles. Precisely, spectral power was estimated for frequency bins of 1 Hz with a 0.1 Hz resolution. At each bin, a window size was set so it contained 20 full cycles of the slowest frequency component (i.e., 20-sec for 1–2 Hz, 10-sec for 2–3 Hz, 6.67-sec for 3–4 Hz, and so on), and step size was set to yield 87.5% overlap between consecutive windows. The number of 20 cycles was chosen so it provided sufficient data for power spectrum reconstruction even at higher frequencies (i.e., 0.45-sec for 44–45 Hz). Given its confirmatory nature, this analysis was only performed for mixed/raw power. At each window-frequency bin pair, power was first computed via the Welch periodogram method at 0.1 Hz resolution, averaged over the frequency bin, then log-transformed, and finally the mean and variance were computed for each frequency over the available windows. Note that while this approach resolves the inconsistent representation of different frequency components within the time window, it yields a lower number of time estimates for slower components (i.e., 5 for 1 Hz, 17 for 2 Hz, 29 for 3 Hz, and so on) as a tradeoff. Between-group differences for both baseline and fluctuation were assessed for global averages (over all channels) as described previously for Frequency-wise Analysis.

Finally, to explore the generalizability of the observed EEG patterns in our sample, we also analyzed an independent dataset made available by Olejarczyk & Jernajczyk [47]. This dataset contains resting-state EEG recordings from 14 patients with paranoid SZ (7 females aged 28.3 ± 4.1 years and 7 males aged 27.9 ± 3.3 years) and 14 age- and sex-matched HC individuals (7 females aged 28.7 ± 3.4 years and 7 males aged 26.8 ± 2.9 years). EEG was collected from 19 cortical locations according to a standardized 10–20 montage, sampled at 250 Hz. The published data was band-pass filtered with a high-pass filter at 1 Hz and a low-pass filter at 45 Hz. This ultimately reduced the available frequency range for IRASA decomposition with the current parameter settings, as applying a resampling with factor *h* = 2.6 would have introduced the filtering effects at about 20 Hz [37]. Therefore, in these analyses we limited our scope on $\sigma (\alpha {{BLP}}_{{mixd}}^{{EC}})$ and $\sigma (\beta {{BLP}}_{{mixd}}^{{EC}})$, but also carried out surrogate data testing as described previously. Additionally, clinical (PANSS scores) and demographic data was unavailable for this sample, preventing us to replicate our complete analysis pipeline.

## Results

### Global power spectral patterns

**Fig. 2:**
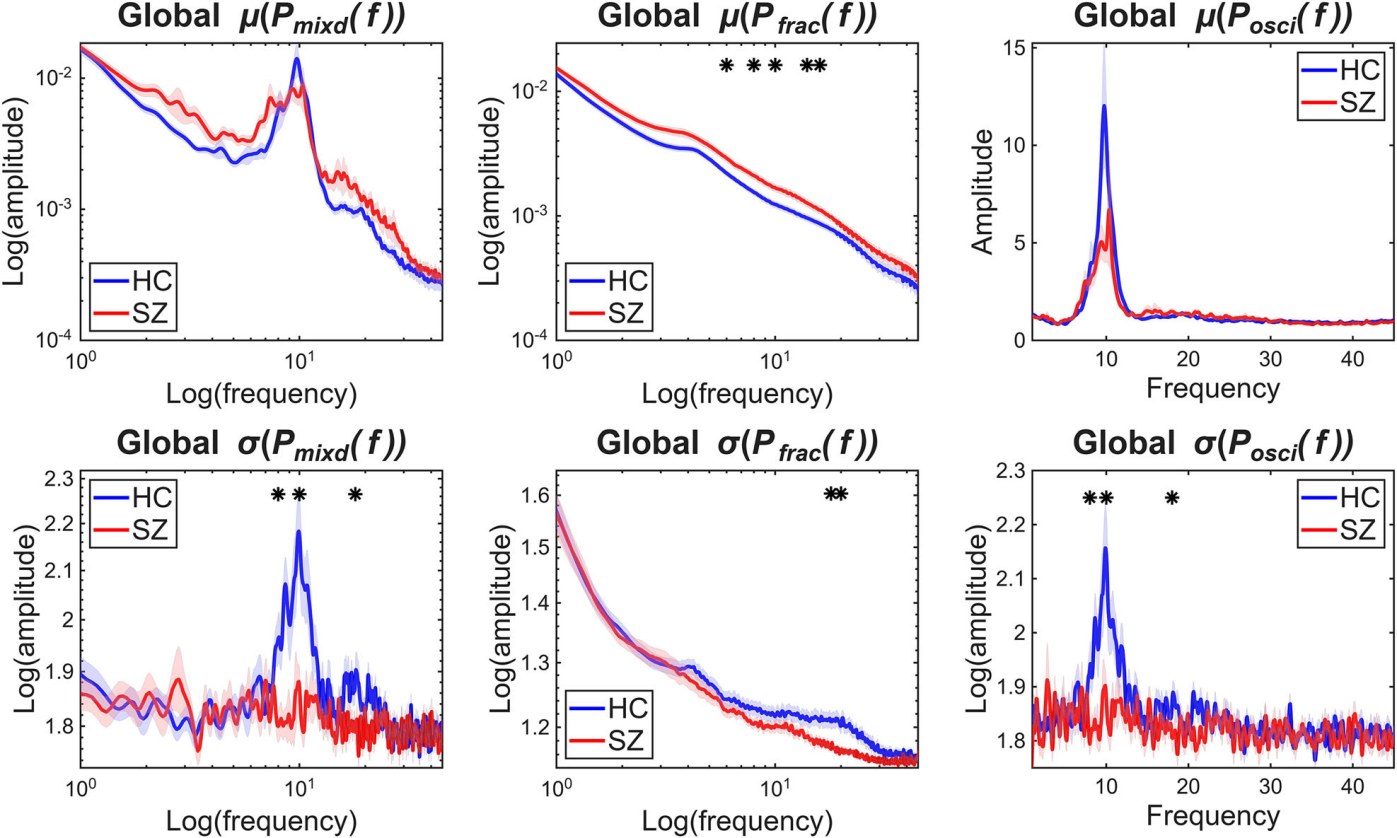
Grand average power spectra taken in eyes-closed (EC) resting state for raw/mixed (left), isolated fractal (middle) and oscillatory (right) components. Top and bottom rows present the average and standard deviation of spectral power taken over sliding windows. Thick lines denote group means, while shaded areas illustrate standard error of the mean. Asterisk symbols indicate significant between-group difference (*p* < 0.05) for each 2-Hz frequency bin. BLP band-limited power, HC healthy control, SZ schizophrenia.

Grand average spectra illustrating baseline power are presented in the upper panels of Fig. 2. We found more power distributed to lower frequencies in SZ compared to HC, although this did not reach statistical significance after adjusting for multiple comparisons. However, increased $\mu ({P}_{{frac}}\left(f\right))$ was found in SZ compared to HC between 5–11 Hz and 13–17 Hz. Regarding temporal variance in spectral power (Fig. 2, lower panels), SZ patients expressed reduced $\sigma ({P}_{{mixd}}\left(f\right))$ and $\sigma ({P}_{{osci}}\left(f\right))$ between 7–11 Hz and 17–19 Hz, while reduced $\sigma ({P}_{{frac}}\left(f\right))$ in the 17–21 Hz range.

**Fig. 3:**
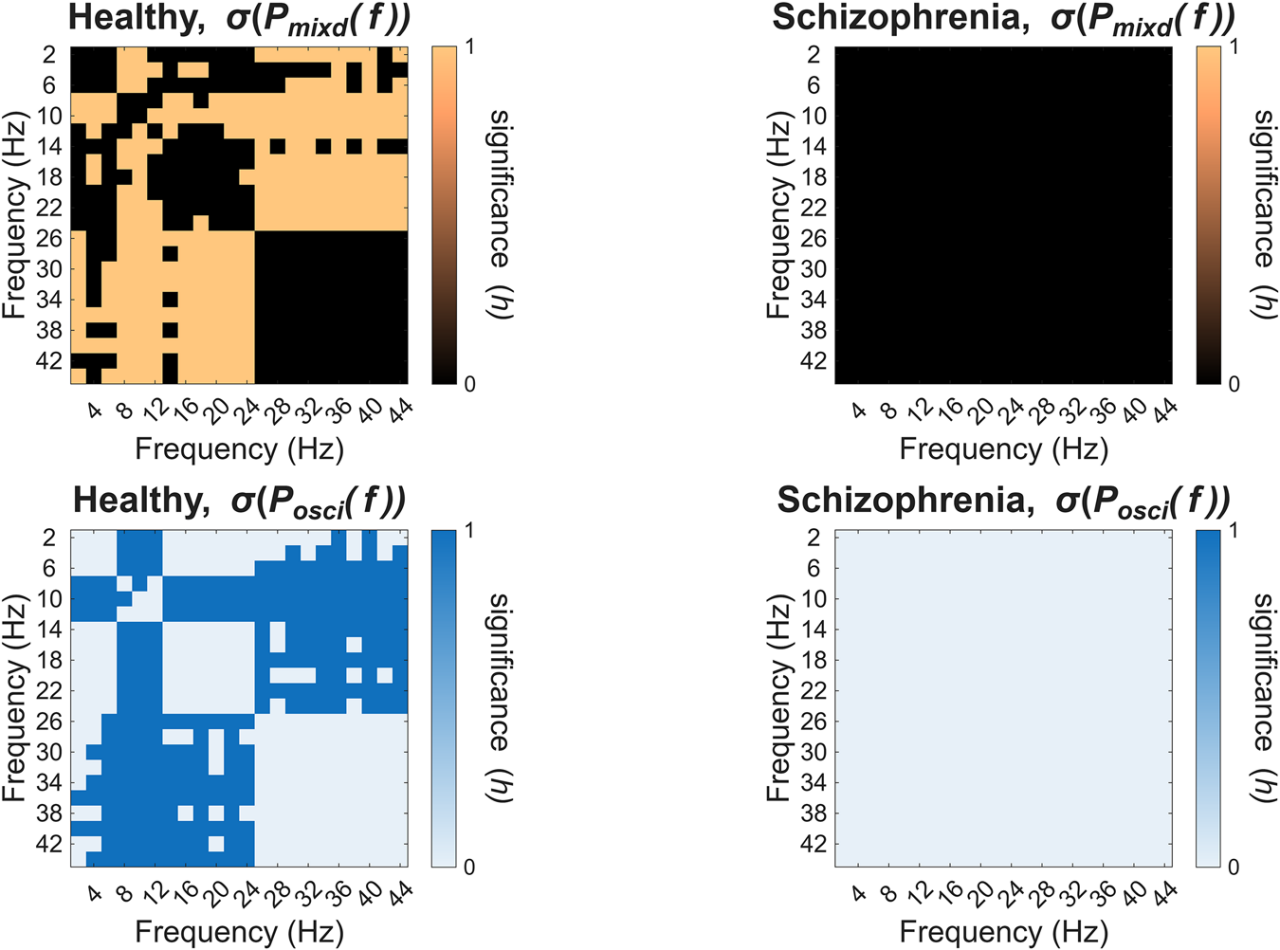
Within-group analysis of power spectral variance in the Healthy (left) and Schizophrenia cohorts. Matrices present hypothesis testing outcomes of bin-to-bin comparisons for raw/mixed (upper) and oscillatory (lower) spectral power in the 1–45 Hz regime, where 1 and 0 denote significant between-bin difference and no difference, respectively. For both analyses, no significant differences were observed in the schizophrenia group, indicating similar time-variance at all frequencies.

While in the HC group $\sigma ({P}_{{mixd}}\left(f\right))$ and $\sigma ({P}_{{osci}}\left(f\right))$ was higher in regimes roughly corresponding to the alpha and beta bands, in the SZ group variance was instead distributed evenly in the 1–45 Hz range. This was explicitly tested via within-group analyses, as shown in Fig. 3. It can be observed that while in the HC group $\sigma ({P}_{{mixd}}\left(f\right))$ and $\sigma ({P}_{{osci}}\left(f\right))$ in the 7–13 Hz regime was significantly higher compared to most other frequencies, no significant bin-to-bin differences were identified in the SZ group for either EEG metrics.

In summary, these results indicate elevated baseline fractal activity in SZ, with a simultaneous reduction in the temporal fluctuation of alpha and beta oscillatory components.

### Power spectral dynamics and topology

**Fig. 4:**
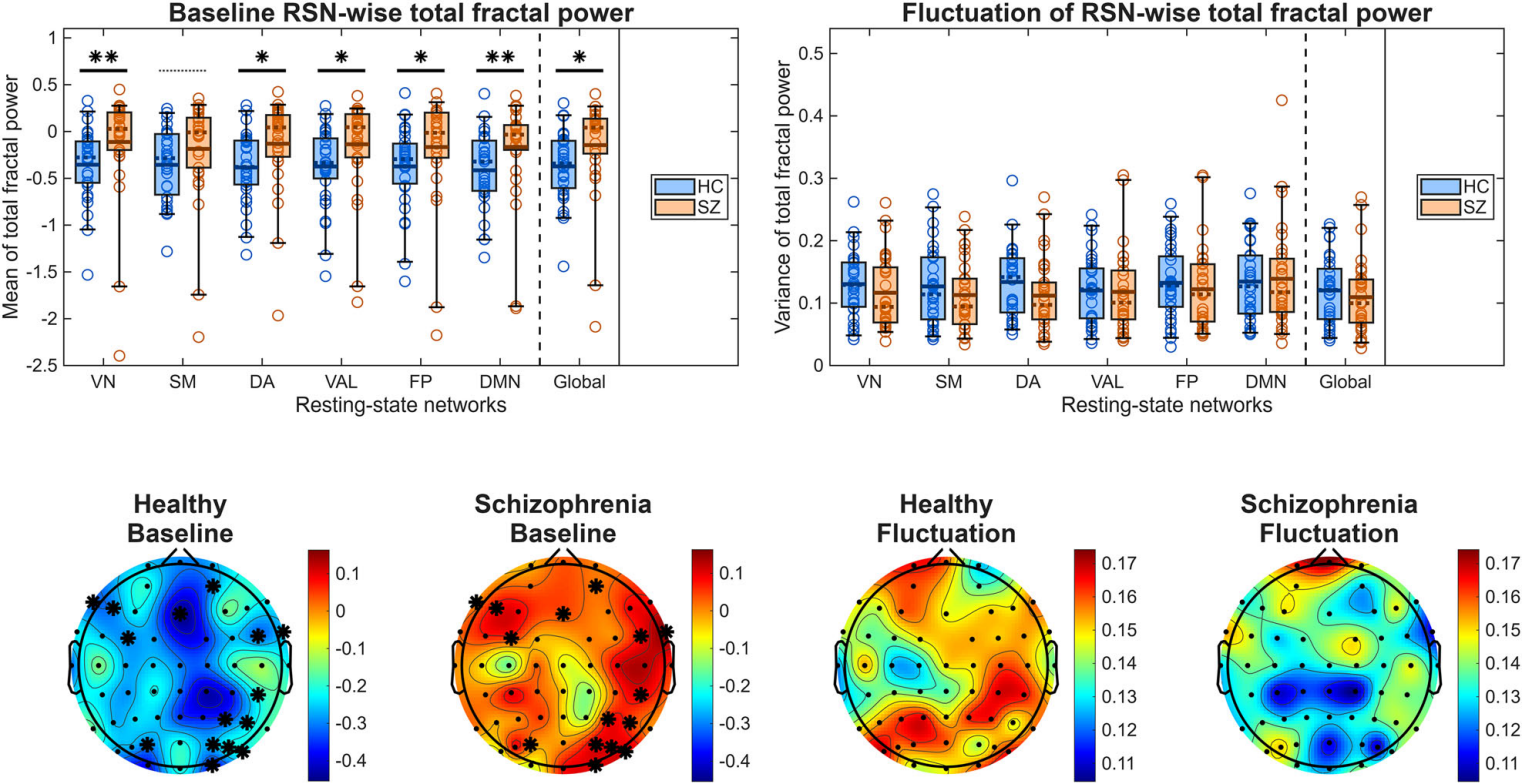
Baseline (left) and fluctuation (right) of total fractal power characteristics in healthy controls and schizophrenia patients. Upper panels show between-group comparisons on the level of resting-state networks (RSNs) as well as whole-cortex average. Box plots show the mean (continuous line), median (dashed line), inter-quartile range (box) and 5^th^ and 95^th^ percentiles (whiskers) along individual data points. Thick horizontal lines indicate significant between-group differences with * and ** denoting *p* < 0.05 and *p* < 0.01, respectively. Dotted lines indicate differences that were rendered non-significant by multiple comparisons adjustment. Lower panels illustrate the scalp topology of corresponding spectral features (baseline: left; fluctuation: right) in the healthy and schizophrenia patient groups. Asterisk symbols denote scalp locations that indicated significant HC vs. SZ difference in the exploratory channel-wise analysis.

Baseline FTP was increased in SZ compared to HC over all RSNs except for the SM network (Fig. 4, left), although fluctuation of FTP indicated no between-group difference (Fig. 4, right).

**Fig. 5:**
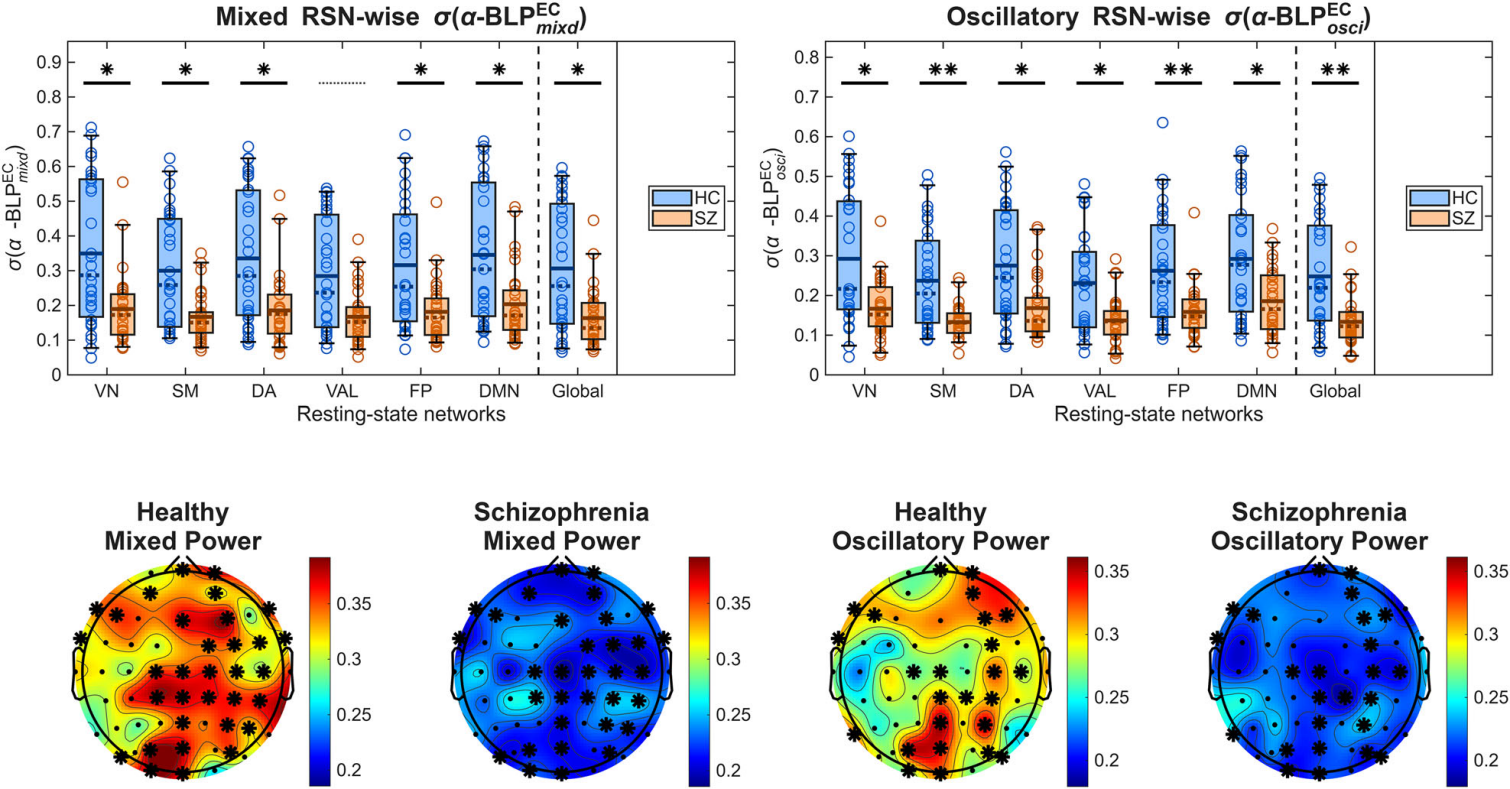
Fluctuation of alpha band-limited power (BLP) as obtained from raw/mixed (left) and isolated oscillatory (right) spectra. Upper panels show between-group comparisons on the level of resting-state networks (RSNs) as well as whole-cortex average. Box plots show the mean (continuous line), median (dashed line), inter-quartile range (box) and 5^th^ and 95^th^ percentiles (whiskers) along individual data points. Thick horizontal lines indicate significant between-group differences with * and ** denoting *p* < 0.05 and *p* < 0.01, respectively. Dotted lines indicate differences that were rendered non-significant by multiple comparisons adjustment. Lower panels illustrate the scalp topology of corresponding spectral features (mixed BLP: left; oscillatory BLP: right) in the healthy and schizophrenia patient groups. Asterisk symbols denote scalp locations that indicated significant HC vs. SZ difference in the exploratory channel-wise analysis.

There was a ubiquitous reduction of $\sigma (\alpha {{BLP}}_{{mixd}}^{{EC}})$ in SZ compared to HC (Fig. 5, left), identified as statistically significant over all RSNs (including global) except the VAL network. The IRASA decomposition revealed that this difference could be attributed to oscillatory activity (Fig. 5, right), with lower $\sigma (\alpha {{BLP}}_{{osci}}^{{EC}})$ in SZ over all RSNs and in global average.

**Fig. 6:**
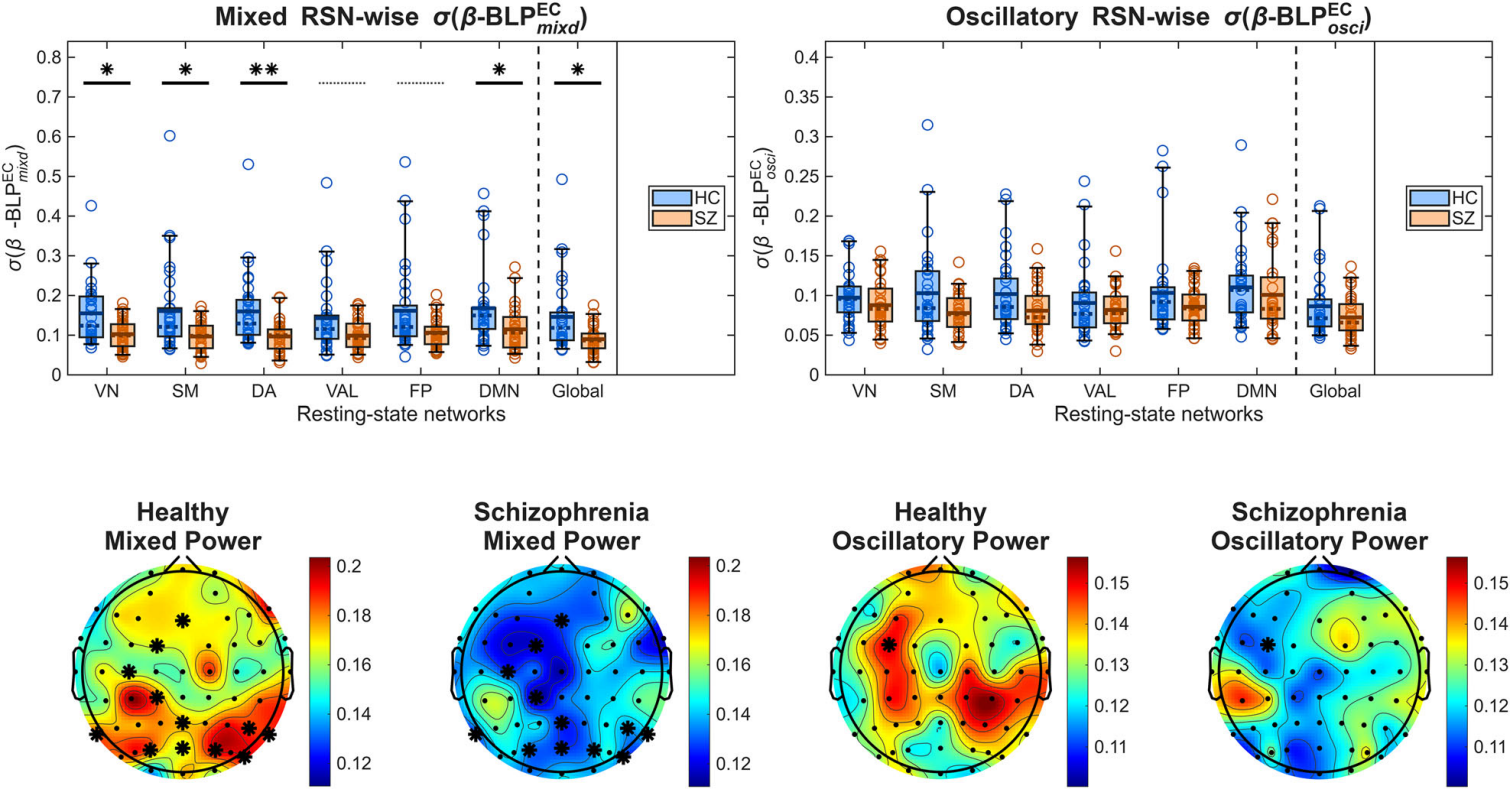
Fluctuation of beta band-limited power (BLP) as obtained from raw/mixed (left) and isolated oscillatory (right) spectra. Upper panels show between-group comparisons on the level of resting-state networks (RSNs) as well as whole-cortex average. Box plots show the mean (continuous line), median (dashed line), inter-quartile range (box) and 5^th^ and 95^th^ percentiles (whiskers) along individual data points. Thick horizontal lines indicate significant between-group differences with * and ** denoting *p* < 0.05 and *p* < 0.01, respectively. Dotted lines indicate differences that were rendered non-significant by multiple comparisons adjustment. Lower panels illustrate the scalp topology of corresponding spectral features (mixed BLP: left; oscillatory BLP: right) in the healthy and schizophrenia patient groups. Asterisk symbols denote scalp locations that indicated significant HC vs. SZ difference in the exploratory channel-wise analysis.

On the other hand, reduced $\sigma (\beta {{BLP}}_{{mixd}}^{{EC}})$ in SZ was more concentrated over posterior and midline scalp locations (Fig. 6, left); this difference was statistically significant over VN, SM, DA and DMN, as well as globally. In contrast to the alpha band, however, IRASA decomposition revealed that this pattern could not be identified in isolated oscillatory power (Fig. 6, right).

Overall, these outcomes indicate characteristic patterns that are distinct for fractal and oscillatory activity. The SZ cohort could be characterized by a widespread increase in TFP, although the time-variability of fractal activity was comparable among HC and SZ. On the other hand, no between-group differences were found in baseline oscillatory alpha activity, but $\sigma (\alpha {{BLP}}_{{osci}}^{{EC}})$ was reduced in the patient group over the entire cortex. Finally, while $\sigma (\beta {{BLP}}_{{mixd}}^{{EC}})$ was reduced in SZ mostly over posterior regions, IRASA decomposition indicated that this was not a result of altered oscillatory activity. Details of the statistical analyses regarding the outcomes detailed above are presented in Supplementary Table S2. Additionally, please note that details and outcomes of all 154 RSN-wise, and 1210 exploratory channel-wise hypothesis tests are provided in the **Online Supplement** (see **Data Availability Statement**).

### Relationship between alpha power and spectral slope

As both alpha BLP and spectral slope was formerly linked to the E/I ratio as its potential EEG markers [15, 44], we also assessed their potential relationship in our sample via LME modeling. This revealed a very strong, positive association between $\alpha {{BLP}}_{{osci}}^{{EC}}$ and ${\beta }_{{hi}}$ in both HC (*r* = 0.6657, *p* < ${10}^{-5}$) and SZ (*r* = 0.5059, *p* < ${10}^{-5}$) groups, indicating that stronger $\alpha {{BLP}}_{{osci}}^{{EC}}$ predicated higher ${\beta }_{{hi}}$ (i.e., steeper spectral slope) and vice versa (Supplementary Figure S1).

### Spectral features and clinical symptoms

**Fig. 7:**
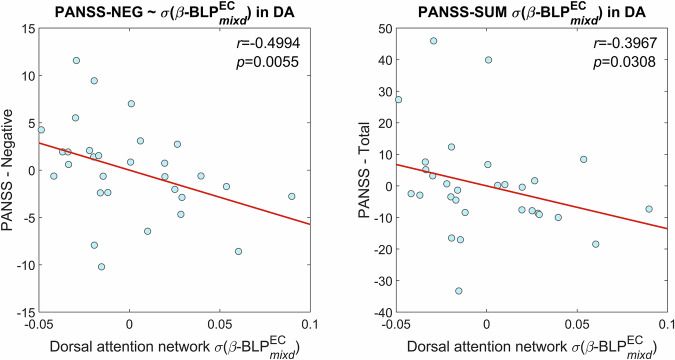
Correlation analysis between $\sigma (\beta BL{P}_{mixd}^{EC})$ and PANSS scores in the Dorsal Attention (DA) network. The neural index was found significantly correlated (*p* < 0.05) with negative (left) and total (right) PANSS scores. All variables were adjusted for potential confounding effects of age, sex, years in education and disease duration. PANSS: positive and negative syndrome scale; NEG: negative; SUM: all symptoms combined.

Correlation analysis revealed significant associations between EEG indices and clinical symptoms in only two cases (Fig. 7), both regarding $\sigma (\beta {{BLP}}_{{mixd}}^{{EC}})$. Precisely, $\sigma (\beta {{BLP}}_{{mixd}}^{{EC}})$ over DA was inversely correlated to PANSS-NEG (Spearman *r* = −0.4994, *p* = 0.0055) and to PANSS-SUM (Spearman *r* = −0.3967, *p* = 0.0308), indicating that higher PANSS scores were associated with greater reduction in temporal variability in beta BLP. Note that these outcomes were not adjusted for multiple comparisons. Detailed reports on all correlation analyses are provided in the **Online Supplement**.

To further explore the potential confounding effects of antipsychotic medication, we correlated global EEG indices with CPZ equivalent dose values. This analysis revealed a positive correlation between CPZ and $\sigma (\beta {{BLP}}_{{mixd}}^{{EC}})$ (Spearman *r* = 0.4399, *p* = 0.0150, unadjusted). Additionally, correlation analysis between CPZ doses and PANSS scores indicated a significant positive correlation (Pearson *r* = 0.3786, *p* = 0.0391, unadjusted) between CPZ and PANSS-NEG (Supplementary Figure S2).

### Testing for nonlinearity

**Fig. 8:**
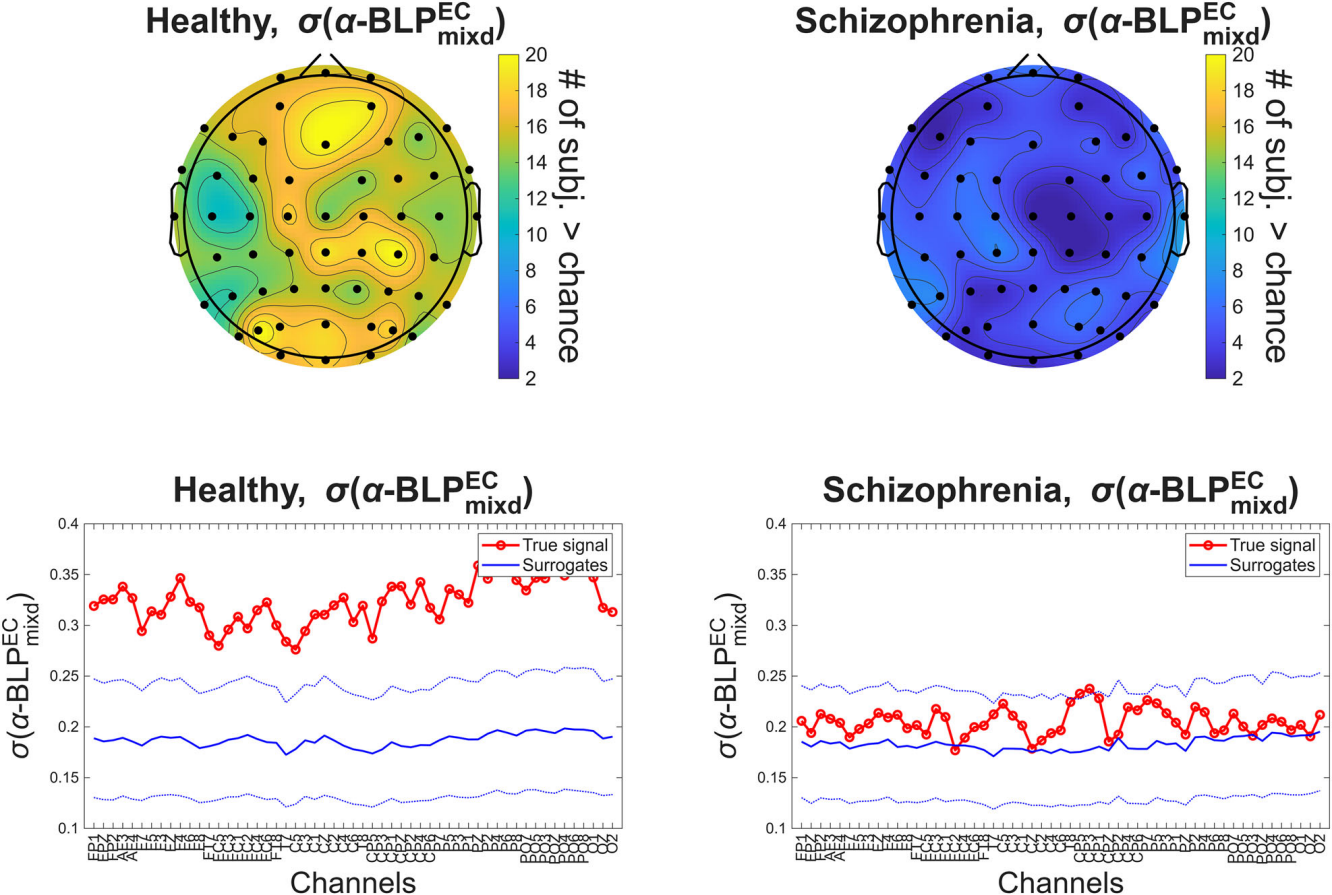
Results of surrogate data analysis in HC (left) and SZ (right) groups in the alpha band (8–13 Hz). The top panels illustrate for every cortical location the number of participants in the respective groups where nonlinearity was confirmed. In the HC group nonlinearity could be confirmed for most locations for at least 15 out of 31 participants, while this proportion was mostly 4–6 out of 30 in SZ. Lower panels show the actual group averages for ${\boldsymbol{\sigma }}({\boldsymbol{\alpha }}{{\boldsymbol{BLP}}}_{{\boldsymbol{mixd}}}^{{\boldsymbol{EC}}})$ in red values obtained from surrogate time series indicated in blue. Continuous blue line denotes the mean from surrogates, while dotted line denotes ± standard deviation from the mean.

Surrogate data testing was utilized to test if fluctuations in BLP were of nonlinear origin. The outcomes of this analysis regarding $\sigma (\alpha {{BLP}}_{{mixd}}^{{EC}})$ are presented on Fig. 8, while those for $\sigma (\beta {{BLP}}_{{mixd}}^{{EC}})$ are on Supplementary Figure S3. Locations where nonlinearity was confirmed for higher proportions (i.e., >15 subjects) are mostly located over fronto-central and parieto-occipital regions (Fig. 8, upper panels). On the group level, $\sigma (\alpha {{BLP}}_{{mixd}}^{{EC}})$ was well above values from surrogate data in the HC group, while the obtained values are mostly within the spread of surrogate values in the SZ group (Fig. 8, lower panels). Similar patterns are observed for $\sigma (\beta {{BLP}}_{{mixd}}^{{EC}})$ (Supplementary Figure S3).

Furthermore, between-group analyses indicated that previously observed differences – reduced $\sigma (\alpha {{BLP}}_{{mixd}}^{{EC}})$ and $\sigma (\beta {{BLP}}_{{mixd}}^{{EC}})$ in SZ – were eliminated in phase-randomized surrogates (see the **Online Supplement** for details). In sum, these analyses strongly imply that healthy alpha and beta BLP dynamics are governed by nonlinear phenomena that appear mostly absent in the patient cohort, indicating abnormal and close-to-random phase dynamics in SZ.

### Effect of window size on dynamic estimates

Frequency-wise analysis outcomes at 4- and 2-second window sizes are presented on Supplementary Figures S4–S7. These are in line with those shown in Fig. 2 and Fig. 3, indicating increased baseline fractal power but reduced oscillatory variance in the alpha and beta regimes in SZ. Note, however, that while BLP- and RSN-wise analyses yielded the same overall patterns, these outcomes were rendered non-significant by FDR adjustment at $\alpha =0.05$.

Analysis outcomes when enforcing an equal number of phase cycles per frequency in the sliding windows are presented on Supplementary Figure S8. While only considered for raw/mixed spectral power, this analysis produced equivalent results to those presented at fixed window lengths. Namely, while baseline broadband power exhibited a higher tendency in SZ these differences did not reach the level of significance, while temporal variance in corresponding to the alpha and beta regimes indicated decreased fluctuation in the SZ group.

In sum, both auxiliary analyses confirmed the same overall patterns and between-group differences as observed in the original evaluation pipeline; however, BLP- and RSN-wise outcomes showed lower sensitivity and therefore those results should be considered as exploratory. Note that details of all statistical evaluations from these pipelines are provided in tabular format the **Online Supplement**.

### Confirmatory analysis on independent sample

Due to characteristics of the independent EEG dataset [47] as described previously, we only replicated our analyses regarding mixed power spectra on the global level. This analysis did not indicate a significant difference between HC and SZ (Supplementary Figure S9) or suggested a loss of nonlinearity in the SZ group either in the alpha (Supplementary Figure S10) or beta (Supplementary Figure S11) bands. When comparing data from each group in the database of [47] to their respective match in our dataset, however, both $\sigma (\alpha {{BLP}}_{{mixd}}^{{EC}})$ and $\sigma (\beta {{BLP}}_{{mixd}}^{{EC}})$ in was significantly lower in our patient sample (*p* = 0.036 and *p* = 0.022 for alpha and beta, respectively), while we did not find any differences between our HC cohort and that from the independent dataset. Further details of these analyses are reported in the Supplementary Material. In summary, while we could not reproduce the observed between-group differences on an independent EEG dataset, this was likely due to differences in the two patient cohorts, as patterns of the two control groups were similar.

## Discussion

Our findings demonstrate that a time-resolved approach to BLP analysis in SZ is warranted, with separating aperiodic scale-free and oscillatory EEG spectral components. We found increased baseline fractal power, while decreased time-variability in the alpha and beta bands in SZ. Decomposition of the spectrum indicated that the slow-wave baseline shift and decrease in beta fluctuations was predominantly attributed to broadband fractal activity, while the decrease in alpha fluctuations were more likely of oscillatory origin. Baseline distinguishing EEG features were uncorrelated with disease symptoms, but beta-band dynamics showed associations with clinical presentations (see Fig. 7). These results point towards a general slowing of brain activity resulting in the allocation of more power towards smaller frequencies, with at the same time alpha oscillations and beta activity becoming less variable over time.

### Relationship with E/I ratio

In our previous work [28] we exclusively focused on the fractal component of the power spectra to characterize spectral slopes in 1–4 (${\beta }_{{lo}}$) and 20–45 Hz (${\beta }_{{hi}}$) regimes. That analysis indicated no between-group difference in the temporal average of slopes, however it revealed a reduction in the temporal variance of ${\beta }_{{hi}}$ over the DA network. Notably, resting alpha power is hypothesized to reflect inhibitory tone [43], and thus its changes might also be associated with changes in the E/I ratio [44, 48]. In our current analysis, oscillatory alpha power exhibited similar characteristics, in that baseline was comparable among the two groups, while temporal variability was reduced in the SZ compared to the HC group. These findings are in line with those of Abbasi and colleagues, showing no HC vs. SZ difference in E/I ratio during rest, while increased E/I balance in SZ was only revealed during task conditions [49]. Furthermore, previous research indicates that scale-free (or fractal) neural activity is stable and minimally affected by rest-to-task transitions in contrast to oscillatory activity, which shows flexibility [22]. Along these lines, increased baseline TFP in SZ is plausibly represent the increased level of spontaneous neural activity as previously observed in [49]. In sum, these findings imply that while resting E/I ratio is likely comparable among HC and SZ, the dynamic flexibility of E/I balance indicate disease-related changes, as shown in spontaneous fluctuations and adaptation during task performance.

The LME analysis revealed a strong relationship between $\alpha {{BLP}}_{{osci}}^{{EC}}$ and ${\beta }_{{hi}}$, raising the concern that our previous outcomes were confounded (or driven by) oscillatory alpha instead of broadband scale-free activity. We propose two, not necessarily exclusive explanations for this strong dependence. First, our previous analytical approach utilizing IRASA was not delicate enough to compensate for potential bias of oscillatory alpha activity on the 20–45 Hz range when estimating ${\beta }_{{hi}}$. Even though we defined the specifics of our analytical approach with this concern in mind – i.e., excluding 4–20 Hz regime from slope estimation, setting the rescaling parameter $h$ ranging from 1.1 to 2.6 to avoid leakage and smearing effects [16, 37]–, unfortunately we cannot completely exclude this possibility. On the other hand, alpha activity is broadly considered as a neural signature of inhibitory tone [42, 43]. While also observed in [44], recent studies indicated that dynamics in alpha power are related to E/I ratio: namely, alpha bursts with high amplitude might represent short periods with stronger inhibition, alternating with excitation/disinhibition [50, 51]. These phenomena are well in line with our findings and could provide an explanation on why we see an alignment between $\alpha {BLP}$ and ${\beta }_{{hi}}$. Nevertheless, this problem appears very relevant for studies inferring the E/I ratio from EEG analysis and warrants future research.

### Reduced power fluctuations and loss of nonlinearity

Systems that exhibit power-law ($1/{f}^{\beta }$) scaling as a local instead of global property are referred to as multifractals [52, 53]. In case of temporal processes this means that the fractal scaling exponent $\beta$ changes over time instead of being constant [54–57]. Such phenomena often emerge in biological systems as a result of nonlinear, antagonistic feedback loops such as the net effect of the sympathetic and parasympathetic nervous system [58, 59], or the interaction of excitatory and inhibitory neuronal populations [60]. The temporal variability of ${\beta }_{{hi}}$ therefore implies that the neural processes generating the EEG signal might be multifractal; however, such direct confirmation of multifractality usually demands immense computational capacity [61, 62]. Instead, we decided to test if nonlinearity might explain the emergence in the time-variability of alpha and beta BLP, the former of which we also used as a proxy for ${\beta }_{{hi}}$ given their strong correlation. Surrogate data testing indeed confirmed altered phase dynamics in SZ. This finding falls in line with the recently proposed phase-based temporal imprecision model for psychosis [23, 24], which hypothesizes timing deficits in both phase and amplitude as a potential etiological factor of perceptional disfunctions in SZ. This phenomenon becomes most apparent in reduced intertrial phase coherence in time-locked task paradigms [23]; however, increased randomness of phase dynamics in SZ is also observed in the resting-state [63]. This latter result is directly supported by our findings: while BLP dynamics could not be distinguished from those in phase randomized surrogates in SZ, phase randomization efficiently destroyed fluctuations in alpha and beta BLP in HC.

### Confirmatory analyses

We did not observe the same reduction of $\sigma \left(\alpha {{BLP}}_{{mixd}}^{{EC}}\right)$ in the SZ cohort when analyzing the independent dataset shared by Olejarczyk and Jernajczyk [47]. Notably, estimates obtained in the HC cohorts were comparable, suggesting that potential between-dataset differences are not primarily driven by data collection specifics. There are a couple considerations that might explain these outcomes. Most importantly, patients enrolled in [47] were subjected to a medication washout period of at least seven days before EEG measurement, while our patients were on medication at the time of assessment. This raises the concern that the observed EEG patterns rather reflect response to medication than neural characteristics of the disease, however a recent review addressing pharmacotherapy effects on EEG in SZ does not indicate any previous works reporting changes in short-term alpha- or beta-band BLP dynamics in response to antipsychotic medication [64] and the potential effects of antipsychotics on nonlinear characteristics of EEG are also poorly understood, although some reports might indicate a reduction in nonlinearity in response to antipsychotic medication [65, 66]. It should be noted that the sample in [47] contained only paranoid SZ patients in contrast to our cohort, in which only 7 out of 30 patients were diagnosed as paranoid-type SZ. While previous literature indicates EEG differences in paranoid compared to other subtypes of SZ [67], this is unlikely to explain the differences observed between the two datasets, as EEG indices in our sample were not correlated with positive symptoms. Nevertheless, we must emphasize that our analyses were limited to $\sigma \left(\alpha {{BLP}}_{{mixd}}^{{EC}}\right)$ as we were unable to replicate our exact analytical pipeline due how the shared data was pre-treated (i.e., low-pass filtering at 45 Hz). Therefore, future work on suitable data is required to better address the generalizability of our findings reported here.

### Potential effects of medication

While the positive correlation between CPZ indicates the influence of medication on EEG dynamics, it is also important to highlight that medication was heterogenous in our sample (see Supplementary Table S1) with vastly different mechanisms of action, complicating potential influences on EEG markers [68]. Pharmaceutical therapy is a multifaceted endeavor in SZ, as often more severe symptoms (higher PANSS scores) warrant more potent pharmaceutical intervention, while a more aggressive treatment plan can result in a more successful repression of symptoms (lower PANSS scores). In our sample we found a significant positive correlation between CPZ and PANSS-NEG but no relationship with positive symptoms that can be more efficiently managed by antidopaminergic medication [69]. However, since the relationship between $\sigma (\beta {{BLP}}_{{mixd}}^{{EC}})$ and PANSS-NEG was anticorrelated after controlling for CPZ in both variables, these outcomes suggest that it is unlikely that EEG features and PANSS scores were both vastly confounded by medication and therefore it is probable that these EEG features could indeed reflect pathophysiology and/or therapy response.

### Limitations and future perspectives

The reported EEG patterns in SZ were associated with clinical appearance, and therefore they could potentially be utilized in predicting therapy response and selecting the intervention strategy accordingly, which is still a major challenge for SZ [70]. This calls for prospective, longitudinal studies with only first-episode, drug-naïve SZ participants, so it can be appropriately assessed if the observed patterns are a response to antipsychotic medication, they reflect ongoing pathology, or both. Longitudinal follow-up will also be impediment to evaluating if dynamic spectral features carry any predictive potential in therapeutic strategy planning. Some methodological concerns also need attention. In this work we estimated dynamic properties from only 30 s of EEG data out of the available ~2 min; given the preliminary scope of this study, we prioritized clean, high-quality data, which came at the cost of shorter admissible segments. As our analyses were conducted in the sensor space, topological considerations from the RSN-level findings should be treated as exploratory – especially as we could only replicate these results as tendential at different sliding window sizes –, while future research is warranted using source reconstruction (e.g., [22]) to link the observed patterns to the activity of brain structures. In terms of separating broadband $1/f$ from oscillatory activity in the EEG spectra, following a personalized approach could render our outcomes more relevant on the level of individual patients. A more appropriate characterization of spectral dynamics might be obtained via the Spectral Parametrization (denoted FOOOF) tool of Donoghue and colleagues [19], by taking into consideration the inter-individual variability of peak alpha frequency [71]. In our original analyses we opted to utilize IRASA instead of FOOOF due to the ability of the former to handle multiple 1/f scaling regimes in the fractal component, although recently developed tools might provide additional insight via an adaptive handling of scaling range-dependent spectral bimodality [72]. With present IRASA settings, we could also replicate our analyses on the independent EEG sample only partially. Besides these issues, we also recognize limitations such as low and heterogenous patient sample, lack of explicit measures on other disease features such as cognitive dysfunction [73], and our sole focus on EEG signals over e.g., genetic factors [74]. Importantly, our sample only consisted of resting-state recordings, while including various task states (e.g., auditory oddball) could provide additional insight [21, 22, 49]. Nonetheless, we believe that this study is relevant for drawing attention to the relevance of a dynamic EEG analysis approach in SZ and can lay the foundation for future work overcoming these drawbacks.

### Summary

Our findings indicate that brain activity in SZ is characterized via increased baseline TFP and a widespread reduction in the temporal fluctuation of oscillatory alpha- and total beta-band activity. With regards to the relationships between dynamic EEG markers, symptom scores and medication data, we observed three patterns. First, a negative correlation between EEG markers and PANSS scores. This falls in line with the observed between-group differences (lower time-variability in SZ compared to HC), in that a more extensive loss of temporal BLP fluctuations was associated with more severe clinical symptoms in SZ. Second, there was a positive correlation between the CPZ equivalent dose and dynamic EEG indices, implying that medication in fact increased and not decreased temporal variability in BLP. Therefore, it is unlikely that between-group differences were primarily driven by pharmaceutical effects, even if we failed to replicate this pattern on an independent sample. Third, CPZ was positively correlated with both $\sigma (\beta {{BLP}}_{{mixd}}^{{EC}})$ and PANSS-NEG, and therefore it should not explain the negative correlation between the latter two after controlling for its effect. These outcomes suggest that dynamic EEG indices can indeed be related to disease pathomechanism and/or therapy response in SZ, likely indicating altered phase dynamics and consequent loss of nonlinearity.

## Supplementary information

Supplementary

## Data Availability

The eyes-closed resting-state data analyzed this study has been made available in a public Zenodo repository titled “Resting-state EEG, clinical, and demographics data from schizophrenia patients and age-matched healthy controls” at 10.5281/zenodo.14808295.

## Code availability

All data analyses were carried out via MATLAB (version 2023b) utilizing the EEGLAB toolbox [31] (version 2020.0) along with custom analysis codes. MATLAB codes developed and used for analyzing the data, statistical evaluations (including raw and adjusted *p*-values, test statistic values and effect size measures) and visualizing outcomes (along with pre-processed EEG) are provided in a public GitHub repository at https://github.com/samuelracz/schizophrenia_BLP_dynamics. Note that this **Online Supplement** also contains detailed statistical reports exported in tabular format and also as MATLAB workspaces.
