# Supplementary material for: Diminished variability of alpha and beta band-limited power as a neural signature in schizophrenia
Source: Transl Psychiatry. 2026 May 2;16:315. doi: 10.1038/s41398-026-04055-w (PMC13280145; doi:10.1038/s41398-026-04055-w)
Supplement: Supplementary file 1 — Supplementary [file 41398_2026_4055_MOESM1_ESM.docx]

SUPPLEMENTARY MATERIAL: Diminished Variability of Alpha and Beta Band-limited Power as a Neural Signature in Schizophrenia

Frigyes Samuel Racz, Kinga Farkas, Melinda Becske, Hajnalka Molnar, Zsuzsanna Fodor, Peter Mukli and Gabor Csukly

# Additional Study Cohort Information

All participants in the SZ group had a diagnosis of the condition according to the Diagnostic and Statistical Manual of Mental Disorders, 5th Edition (DSM-5) [1]. Clinical assessment including diagnosis and evaluation of psychiatric symptoms via the PANSS [2] were performed by trained psychiatrists. Exclusion criteria for both groups included history of stroke, intellectual disability, history of epilepsy, substance abuse, and history of head injury with loss of consciousness for over ten minutes. Furthermore, presence or history of any mental disorder according to DSM-5 was an exclusion criterion for HC participants, while presence of an additional condition besides SZ resulted in exclusion from the patient group. Members of the patient cohort were ON medication at the time of EEG recordings and PANSS assessment (**Supplementary Table S1**). General, Negative, Positive and Total PANSS sub-scores in the patient sample were 30.10±9.05, 15.90±5.70, 14.63±5.12 and 60.63±17.74, respectively.

**Supplementary Table S1.** Medication information in the schizophrenia cohort. SZ: schizophrenia; SD: standard deviation.

| **Medication information (SZ group)** | | |
| --- | --- | --- |
| **Medication name** | ***n* (mean dose±SD mg)** | **Medication type** |
| Quetiapine | 3 (500±285 mg) | Antipsychotics |
| Risperidone | 1 (2 mg) |  |
| Flupentixol decanoate | 1 (20 mg) |  |
| Clozapine | 13 (135±132 mg) |  |
| Haloperidol | 3 (3.75±1.1 mg) |  |
| Olanzapine | 4 (8.125±2.4 mg) |  |
| Amisulpride | 5 (430±338 mg) |  |
| Aripiprazole | 8 (16.875±5.3 mg) |  |
| Lithium | 5 (850±224 mg) | Mood stabilizers |
| Valproate | 2 (950±71 mg) |  |
| Lamotrigine | 1 (200mg) |  |
| Clonazepam | 9 (1.5±0.9 mg) | Benzodiazepines |
| Alprazolam | 1 (0.5 mg) |  |
| Paroxetine | 1 (20 mg) | Antidepressants |
| Sertraline | 1 (50 mg) |  |
| Citalopram | 1 (20 mg) |  |

# Details of RSN-wise Analyses

**Supplementary Table S2.** Details of statistical tests at global- and RSN-level comparisons. RSN: resting-state network; ES: effect size.

| **Measure** | **RSN** | **Healthy** | **Schizophrenia** | **Statistic** | ***p*-value** | **ES** |
| --- | --- | --- | --- | --- | --- | --- |
| **Total Fractal Power** | | | | | | |
| $\mu(TFP)$ | Global | -0.3425 [-0.60, -0.10] | 0.0416 [-0.24, 0.14] | *z*=-3.1810 | *p*=0.0015 | \|*r*\|=0.4073 |
| $\mu(TFP)$ | VN | -0.2774 [-0.55, -0.11] | 0.0281 [-0.20, 0.20] | *z*=-3.4551 | *p*=0.0006 | \|*r*\|=0.4424 |
| $\mu(TFP)$ | DA | -0.3784 [-0.57, -0.10] | 0.0432 [-0.27, 0.18] | *z*=-3.0656 | *p*=0.0022 | \|*r*\|=0.3925 |
| $\mu(TFP)$ | VAL | -0.3353 [-0.50, -0.07] | 0.0458 [-0.28, 0.19] | *z*=-3.0512 | *p*=0.0023 | \|*r*\|=0.3907 |
| $\mu(TFP)$ | FP | -0.2957 [-0.56, -0.13] | -0.0138 [-0.28, 0.20] | *z*=-2.8492 | *p*=0.0044 | \|*r*\|=0.3648 |
| $\mu(TFP)$ | DMN | -0.3212 [-0.63, -0.10] | -0.0340 [-0.20, 0.07] | *z*=-3.3108 | *p*=0.0009 | \|*r*\|=0.4239 |
| **Alpha band** | | | | | | |
| $\sigma(\alpha{BLP}_{mixd}^{EC})$ | Global | 0.2557 [0.15, 0.49] | 0.1350 [0.10, 0.21] | *z*=2.9934 | *p*=0.0028 | \|*r*\|=0.3833 |
| $\sigma(\alpha{BLP}_{osci}^{EC})$ | Global | 0.2481 (0.14) | 0.1337 (0.06) | *t_59_*=4.1540 | *p*<0.0001 | *d*=1.0503 |
| $\sigma(\alpha{BLP}_{mixd}^{EC})$ | VN | 0.2868 [0.17, 0.56] | 0.1727 [0.12, 0.23] | *z*=3.1954 | *p*=0.0014 | \|*r*\|=0.4091 |
| $\sigma(\alpha{BLP}_{osci}^{EC})$ | VN | 0.2167 [0.17, 0.44] | 0.1521 [0.12, 0.22] | *z*=2.9790 | *p*=0.0029 | \|*r*\|=0.3814 |
| $\sigma(\alpha{BLP}_{mixd}^{EC})$ | SM | 0.2049 [0.13, 0.34] | 0.1316 [0.11, 0.16] | *z*=3.3685 | *p*=0.0008 | \|*r*\|=0.4313 |
| $\sigma(\alpha{BLP}_{osci}^{EC})$ | SM | 0.2587 [0.14, 0.45] | 0.1507 [0.12, 0.18] | *z*=2.8780 | *p*=0.0040 | \|*r*\|=0.3685 |
| $\sigma(\alpha{BLP}_{mixd}^{EC})$ | DA | 0.2848 [0.17, 0.53] | 0.1754 [0.12, 0.23] | *z*=2.9934 | *p*=0.0028 | \|*r*\|=0.3833 |
| $\sigma(\alpha{BLP}_{osci}^{EC})$ | DA | 0.2448 [0.15, 0.41] | 0.1361 [0.11, 0.19] | *z*=2.9213 | *p*=0.0035 | \|*r*\|=0.3740 |
| $\sigma(\alpha{BLP}_{osci}^{EC})$ | VAL | 0.2281 [0.12, 0.31] | 0.1347 [0.10, 0.16] | *z*=2.7482 | *p*=0.0060 | \|*r*\|=0.3519 |
| $\sigma(\alpha{BLP}_{mixd}^{EC})$ | FP | 0.2623 (0.14) | 0.1586 (0.07) | *t_59_*=3.6512 | *p*=0.0006 | *d*=0.9232 |
| $\sigma(\alpha{BLP}_{osci}^{EC})$ | FP | 0.2540 [0.15, 0.46] | 0.1650 [0.11, 0.22] | *z*=2.9790 | *p*=0.0029 | \|*r*\|=0.3814 |
| $\sigma(\alpha{BLP}_{mixd}^{EC})$ | DMN | 0.3042 [0.17, 0.55] | 0.1711 [0.13, 0.24] | *z*=2.8925 | *p*=0.0038 | \|*r*\|=0.3703 |
| $\sigma(\alpha{BLP}_{osci}^{EC})$ | DMN | 0.2772 [0.16, 0.40] | 0.1657 [0.12, 0.25] | *z*=2.7626 | *p*=0.0057 | \|*r*\|=0.3537 |
| **Beta band** | | | | | | |
| $\sigma(\beta{BLP}_{mixd}^{EC})$ | Global | 0.1185 [0.09, 0.16] | 0.0866 [0.07, 0.10] | *z*=3.0656 | *p*=0.0022 | \|*r*\|=0.3925 |
| $\sigma(\beta{BLP}_{mixd}^{EC})$ | VN | 0.1239 [0.09, 0.20] | 0.0995 [0.07, 0.13] | *z*=3.0512 | *p*=0.0023 | \|*r*\|=0.3907 |
| $\sigma(\beta{BLP}_{mixd}^{EC})$ | SM | 0.1210 [0.10, 0.17] | 0.0985 [0.07, 0.12] | *z*=2.8780 | *p*=0.0040 | \|*r*\|=0.3685 |
| $\sigma(\beta{BLP}_{mixd}^{EC})$ | DA | 0.1293 [0.10, 0.19] | 0.1001 [0.07, 0.11] | *z*=3.3685 | *p*=0.0008 | \|*r*\|=0.4313 |
| $\sigma(\beta{BLP}_{mixd}^{EC})$ | DMN | 0.1498 [0.12, 0.17] | 0.1066 [0.07, 0.15] | *z*=2.6616 | *p*=0.0078 | \|*r*\|=0.3408 |

# Confirmatory Analyses on Independent Dataset

Frequency-wise analysis indicated no difference between HC and SZ groups in either baseline or fluctuation of spectral power. Frequency-wise analysis was executed both using an 8-second sliding window with 1-second step size, as well as varying window sizes with 20 cycles at each frequency; outcomes of the latter are illustrated on **Supplementary Figure S9** below. On the level of BLP (8-second sliding window approach), global $\sigma(\alpha{BLP}_{mixd}^{EC})$ in SZ (0.2692±0.0699) was comparable to that in HC (0.2639±0.1766) with no statistically significant difference (*p*=0.9185, $t_{26}$=-0.1033, Cohen's *d*=0.0379). Similar outcomes were obtained for $\sigma(\beta{BLP}_{mixd}^{EC})$, where the HC group (median: 0.1607, IQR: [0.1245; 0.1890]) exhibited similar values to the SZ group (median: 0.1476, IQR: [0.1368; 0.1947]) with no significant difference (*p*=0.7652, *z*=-0.2987, *r*=0.0564). As a comparison, the same analysis on our sample indicated a significant difference between HC and SZ both in global $\sigma(\alpha{BLP}_{mixd}^{EC})$ (HC: median=0.2682, IQR: [0.1888; 0.4628]; SZ: median=0.1901, IQR: [0.1630; 0.2182]; *p*=0.0012, *z*=3.2387, *r*=0.4147) and $\sigma(\beta{BLP}_{mixd}^{EC})$ (HC: median=0.1457, IQR: [0.1211; 0.1646]; SZ: median=0.1269, IQR: [0.1130; 0.1419]; *p*=0.0170, *z*=2.3875, *r*=0.3057). However, when comparing data from each group in the database of [3] to their respective match in our dataset, both $\sigma(\alpha{BLP}_{mixd}^{EC})$ and $\sigma(\beta{BLP}_{mixd}^{EC})$ in our patient sample was significantly lower (*p*=0.0036, *z*= 2.9103, *r*=0.4387 for alpha, *p*=0.0022, *z*=3.0615, *r*=0.4615 for beta) compared to those of Olejarczyk & Jernajczyk, while we did not find a statistical difference between the two HC cohorts in either variables.

# Supplementary Figures


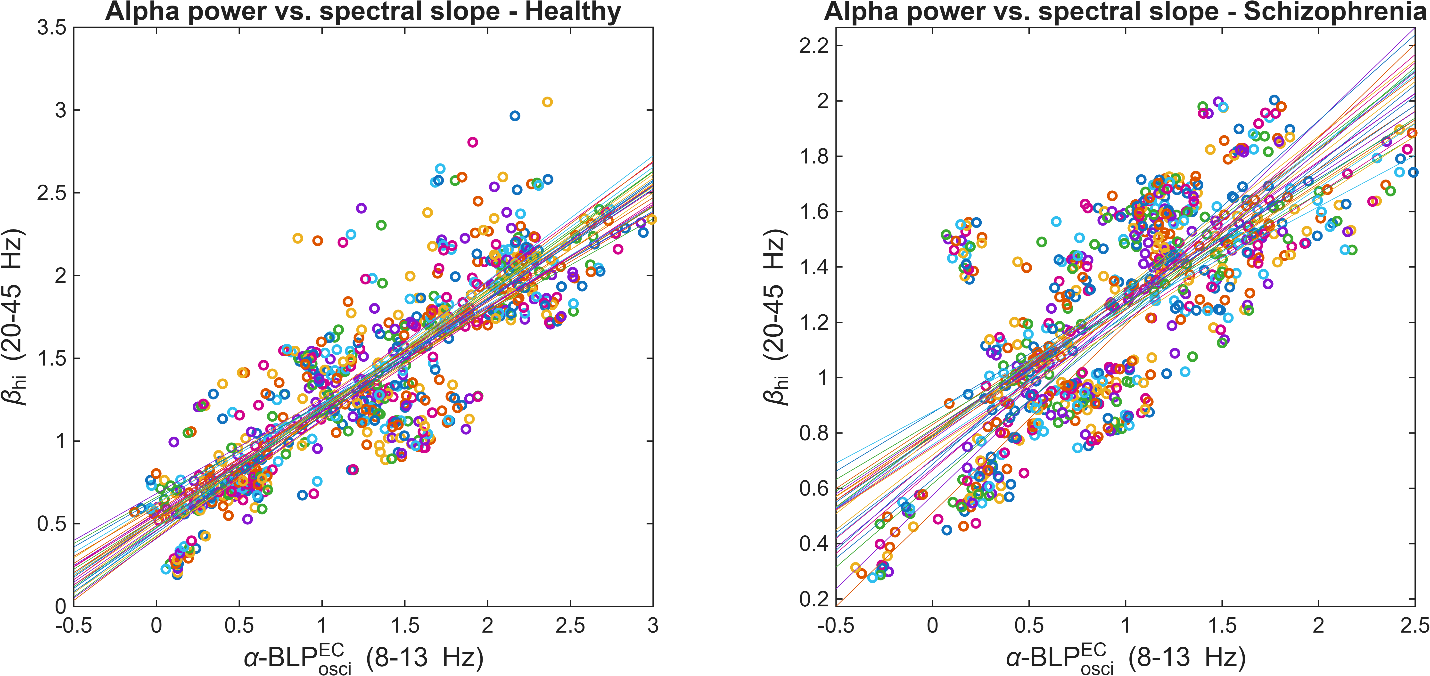


**Supplementary Figure S1.** Illustration of the relationship between $\alpha{BLP}_{osci}^{EC}$ and $\beta_{hi}$ in the healthy (left) and schizophrenia (right) groups. Each circle represents estimates from a single time window (i.e., 23 estimates per subject), with different colors denoting different participant (31 in healthy, 30 in schizophrenia). Straight lines represent the least squares regression fits per subject in matching color. The strong correlation indicates that higher oscillatory alpha power predicates a steeper power spectrum in the 20-45 Hz domain, and this relationship appears similarly in all subjects in both groups.


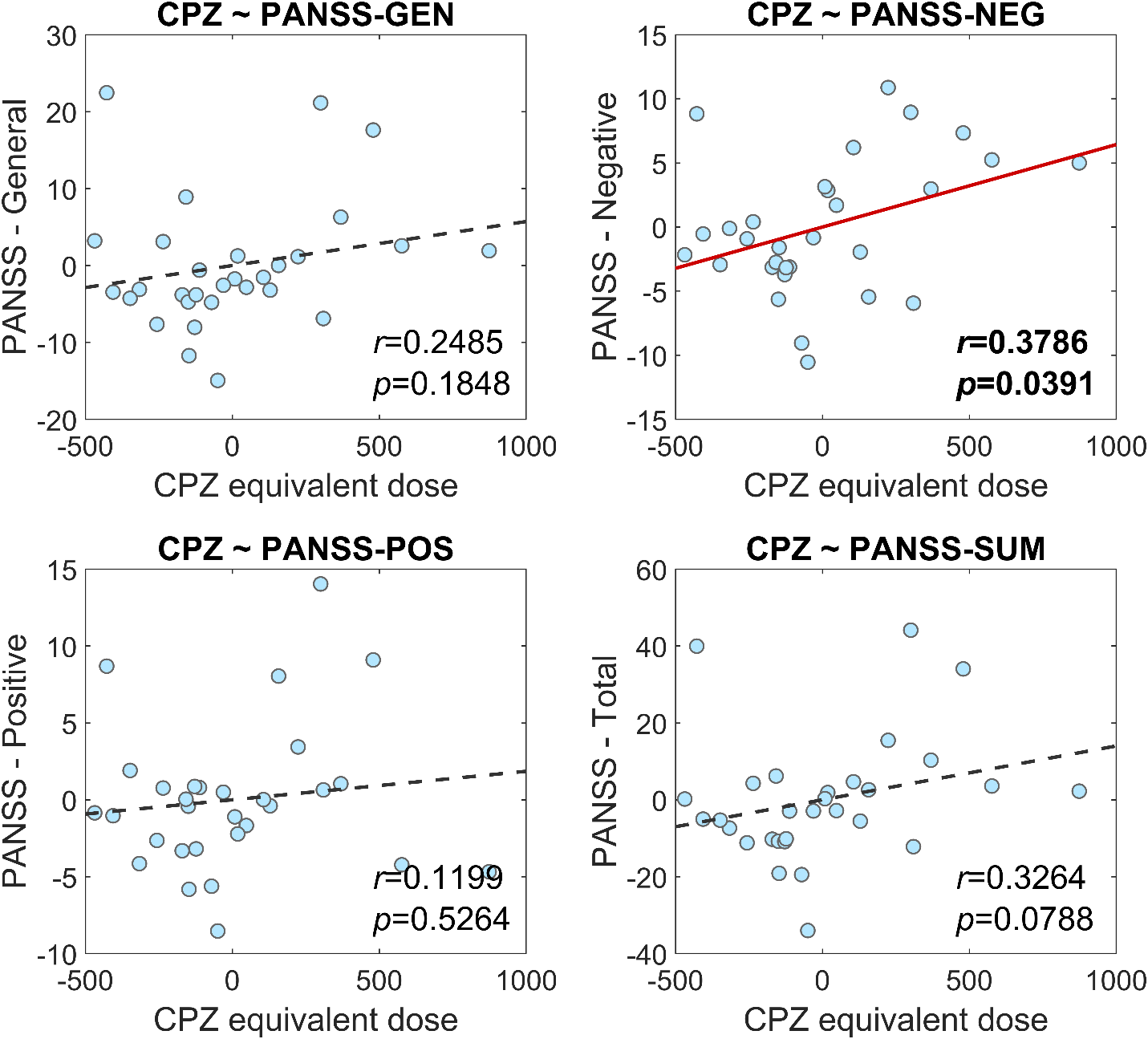


**Supplementary Figure S2.** Correlation analysis between CPZ doses and PANSS scores for general (upper left), negative (upper right), positive (lower left) and all symptoms combined (lower right). A continuous red trend line and bold text indicates significant correlation (p<0.05) between medication and symptoms, while gray dashed line illustrates the trend otherwise. All variables were adjusted for potential confounding effects of age, sex, years in education and disease duration. CPZ: chlorpromazine equivalent dose; PANSS: positive and negative syndrome scale; GEN: general; NEG: negative; POS: positive; SUM: all symptoms combined.


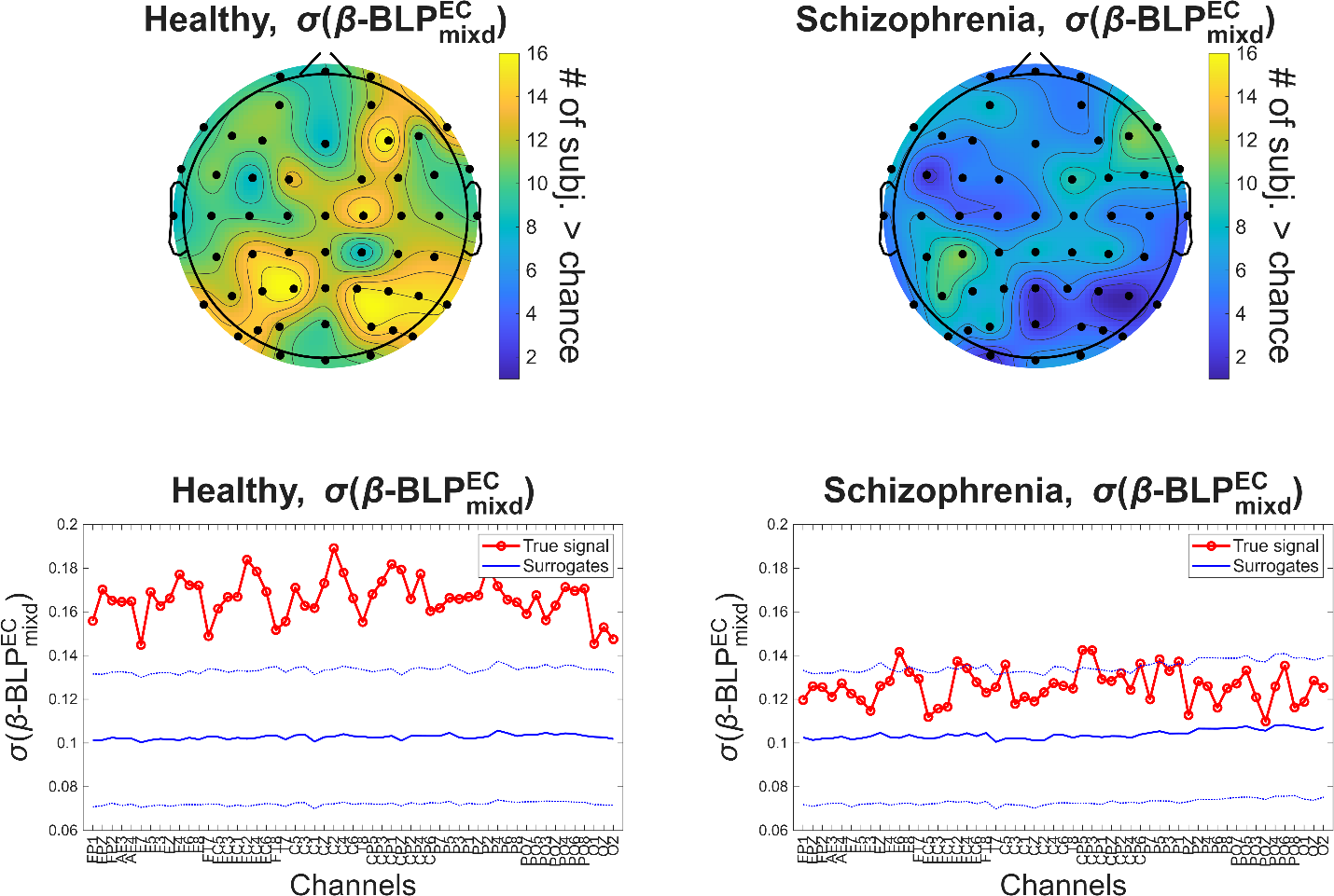


Supplementary Figure S3. Results of surrogate data analysis in HC (left) and SZ (right) groups in the beta band (13-25 Hz). The top panels illustrate for every cortical location the number of participants in the respective groups where nonlinearity was confirmed. In the HC group nonlinearity could be confirmed for most locations for at least 10 out of 31 participants, while this proportion was mostly 4-6 out of 30 in SZ. Lower panels show the actual group averages for $\boldsymbol{\sigma(\beta}\boldsymbol{BLP}_{\boldsymbol{mixd}}^{\boldsymbol{EC}}\boldsymbol{)}$ in red values obtained from surrogate time series indicated in blue. Continuous blue line denotes the mean from surrogates, while dotted line denotes ± standard deviation from the mean.


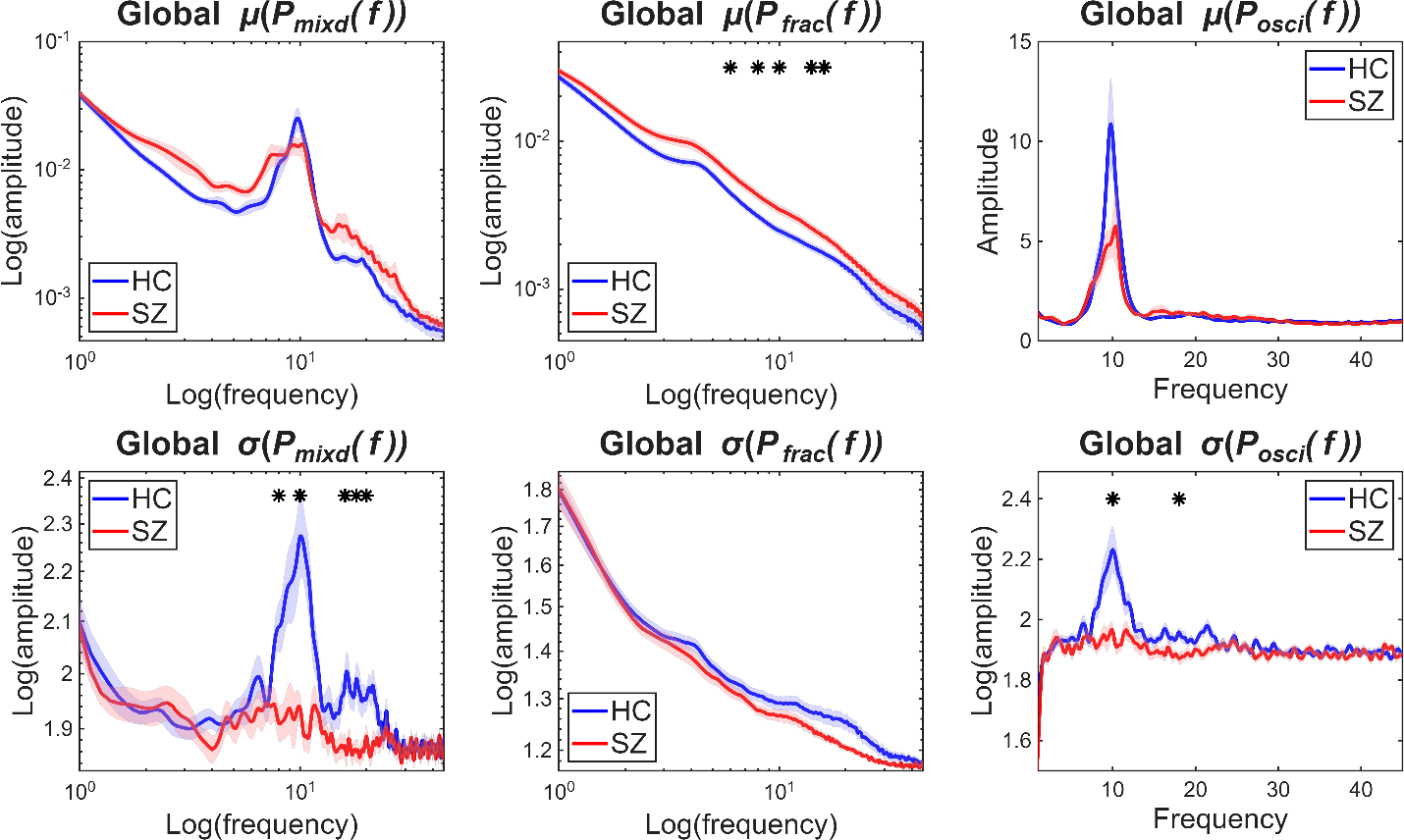


**Supplementary Figure S4.** Grand average power spectra taken in eyes-closed (EC) resting state for raw/mixed (left), isolated fractal (middle) and oscillatory (right) components, computed at 4-second sliding window length. Top and bottom rows present the average and standard deviation of spectral power taken over sliding windows. Asterisk symbols indicate significant between-group difference (p<0.05) for each 2-Hz frequency bin. BLP: band-limited power; HC: healthy control; SZ: schizophrenia.


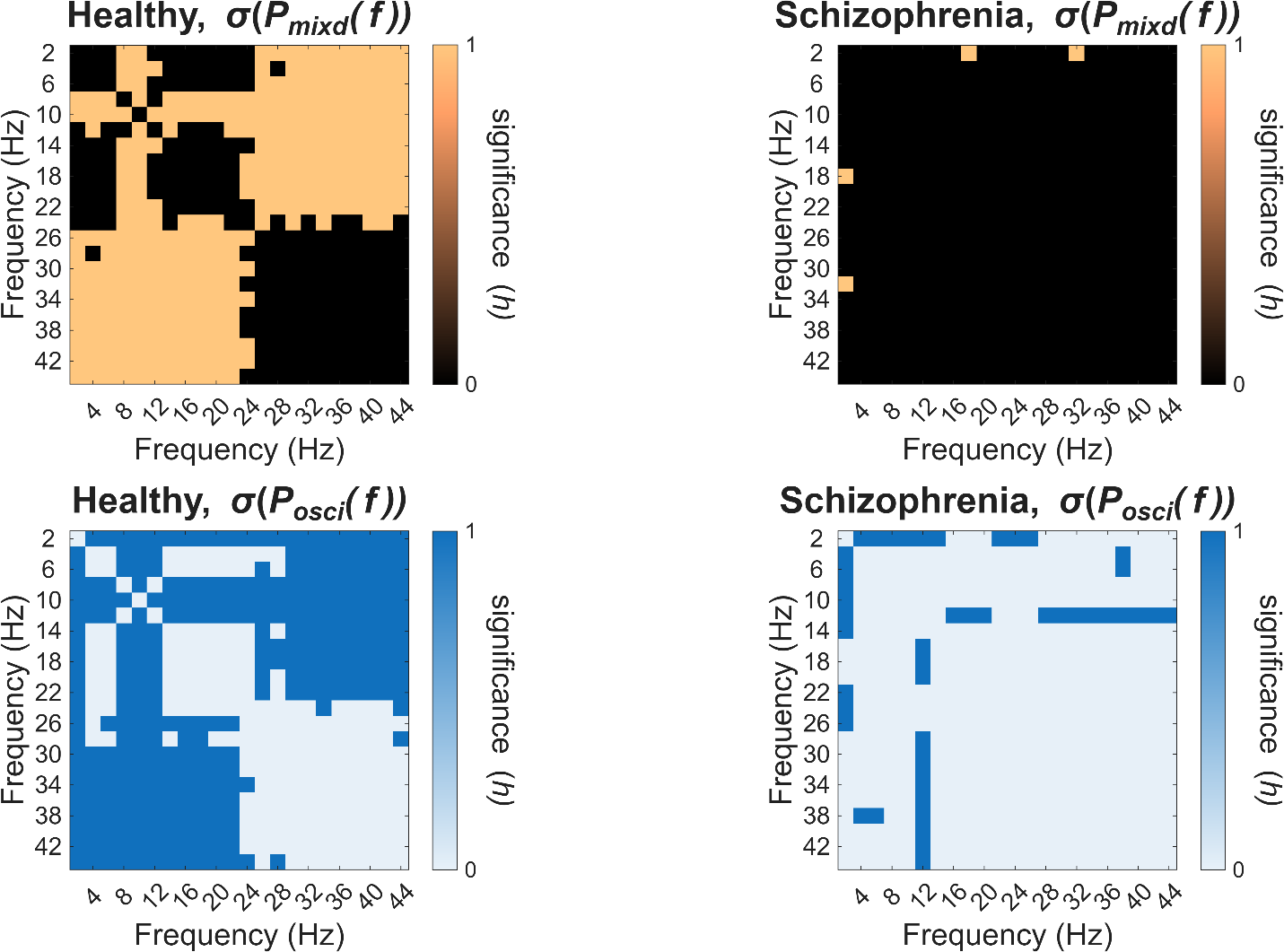


**Supplementary Figure S5.** Within-group analysis of power spectral variance in the Healthy (left) and Schizophrenia cohorts at 4-second sliding window length. Matrices present hypothesis testing outcomes of bin-to-bin comparisons for raw/mixed (upper) and oscillatory (lower) spectral power in the 1-45 Hz regime, where 1 and 0 denote significant between-bin difference and no difference, respectively. For both analyses, significant differences were only observed sporadically in the schizophrenia group, indicating mostly similar time-variance at all frequencies.


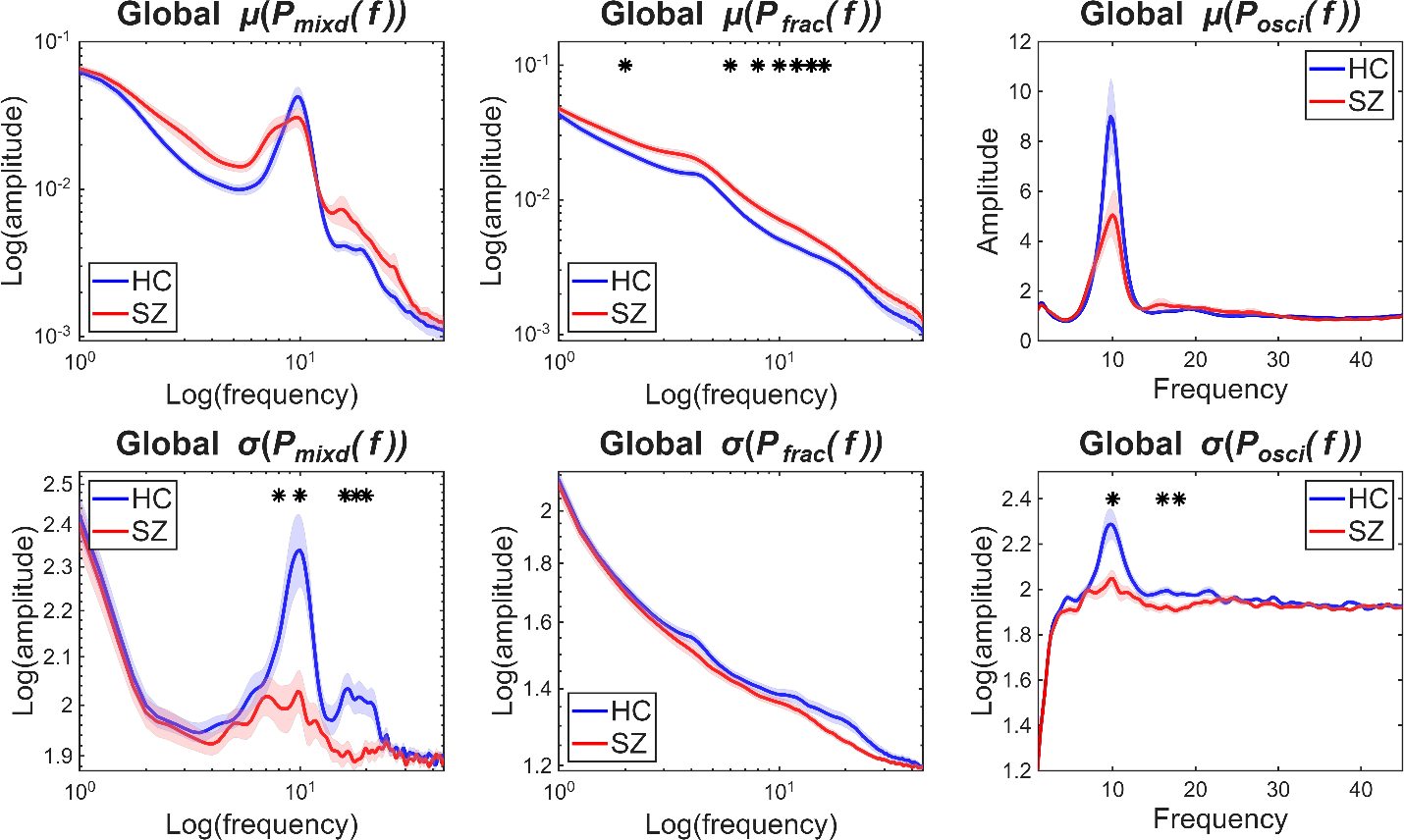


**Supplementary Figure S6.** Grand average power spectra taken in eyes-closed (EC) resting state for raw/mixed (left), isolated fractal (middle) and oscillatory (right) components, computed at 2-second sliding window length. Top and bottom rows present the average and standard deviation of spectral power taken over sliding windows. Asterisk symbols indicate significant between-group difference (p<0.05) for each 2-Hz frequency bin. BLP: band-limited power; HC: healthy control; SZ: schizophrenia.


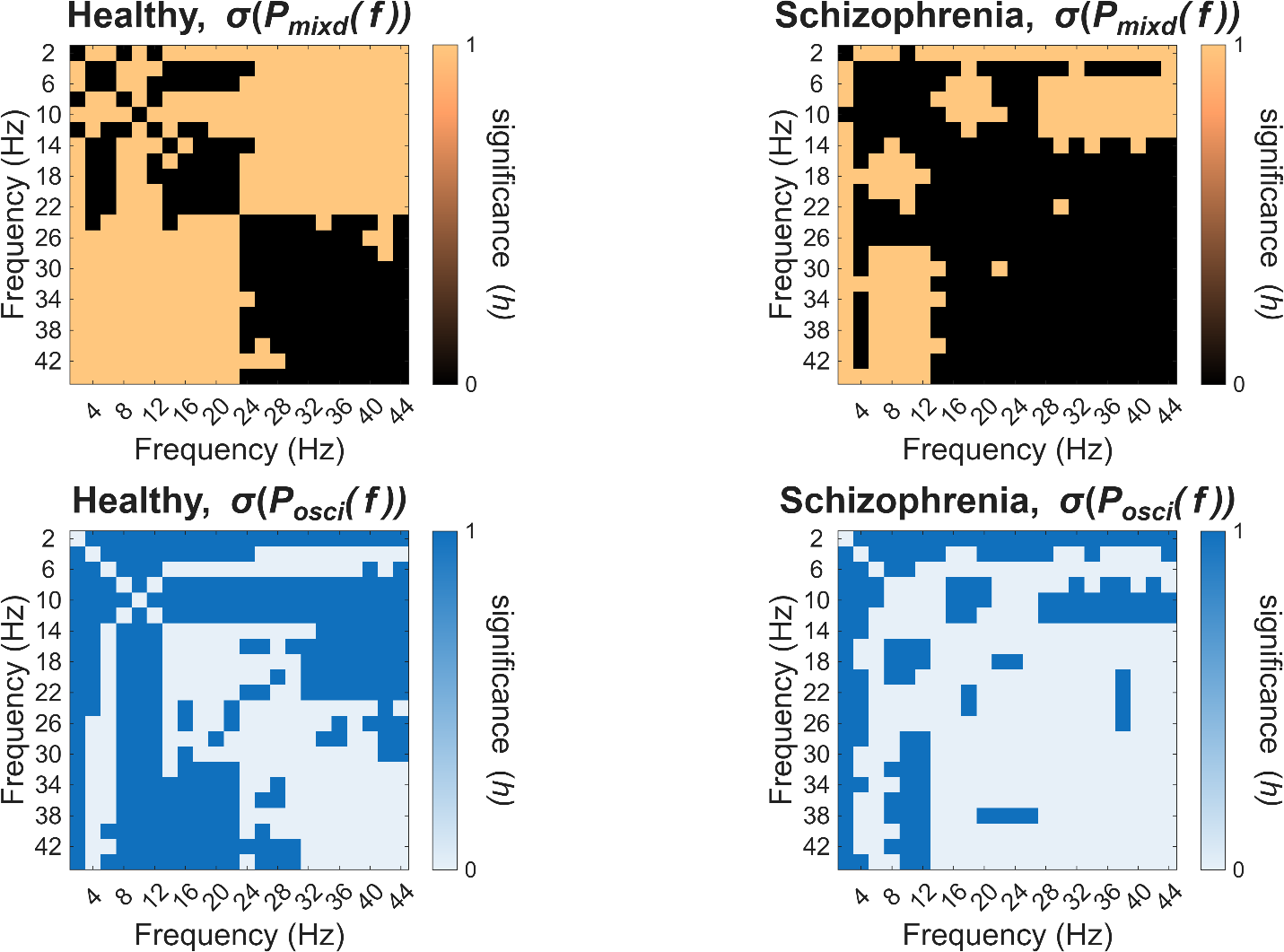


**Supplementary Figure S7.** Within-group analysis of power spectral variance in the Healthy (left) and Schizophrenia cohorts at 2-second sliding window length. Matrices present hypothesis testing outcomes of bin-to-bin comparisons for raw/mixed (upper) and oscillatory (lower) spectral power in the 1-45 Hz regime, where 1 and 0 denote significant between-bin difference and no difference, respectively. For both analyses, significant differences were only observed to a much lesser extent in the schizophrenia compared to the healthy group, indicating more similar time-variance at most frequencies.


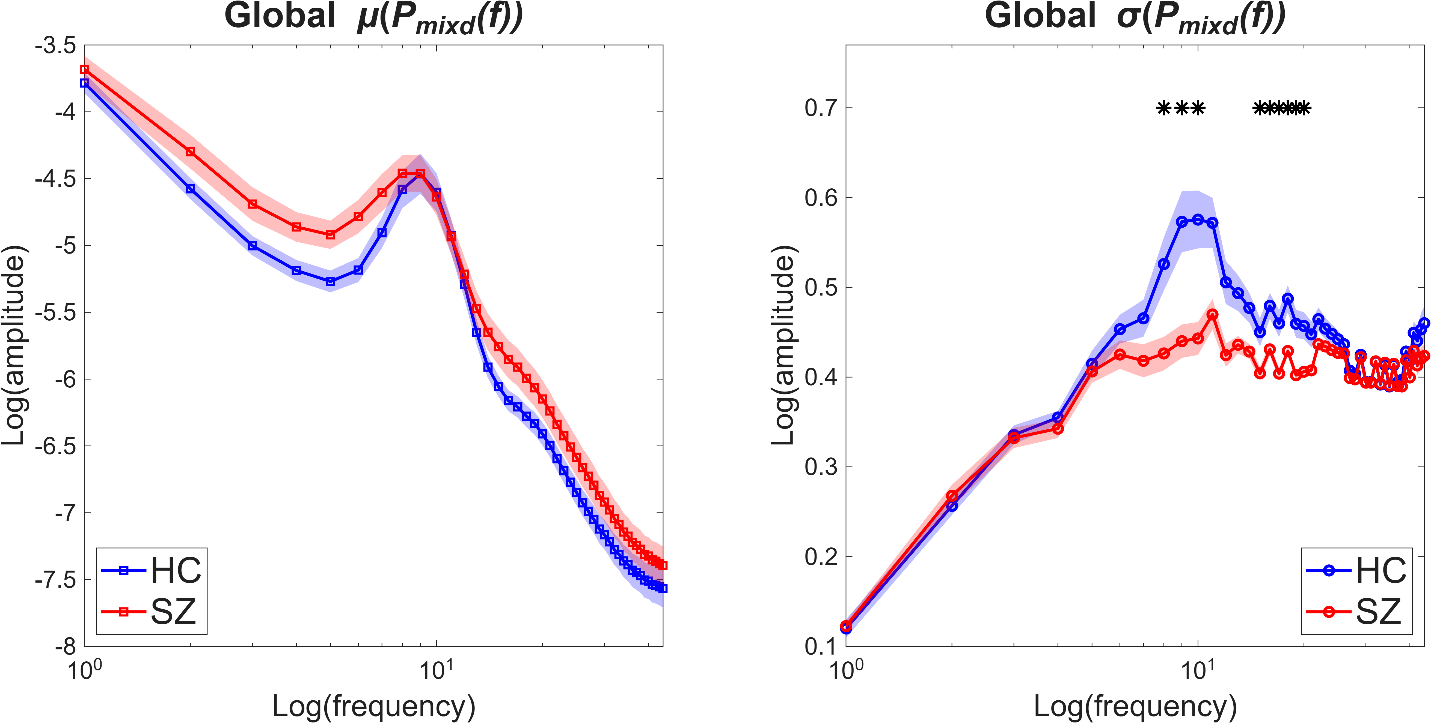


**Supplementary Figure S8.** Grand average power spectra computed at varying window lengths containing an equal number of cycles (20) for all frequencies. The left panel illustrates the baseline, where at each frequency the mean was taken over all available windows. The right panel illustrates fluctuation over time, where at each frequency the standard deviation was taken over all available windows. Asterisk symbols indicate significant between-group difference (p<0.05) after adjusting for multiple comparisons. HC: healthy control; SZ: schizophrenia.


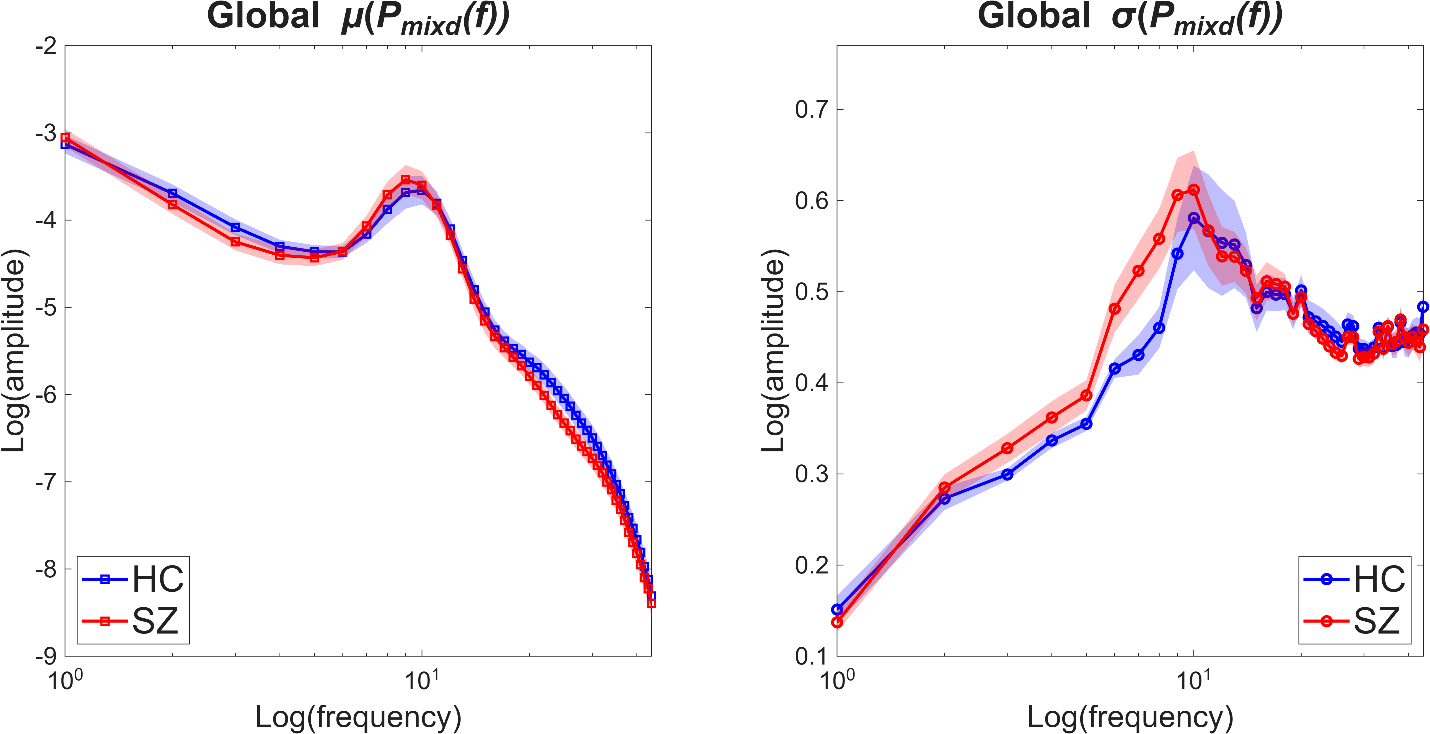


**Supplementary Figure S9.** Grand average power spectra computed at varying window lengths containing an equal number of cycles (20) for all frequencies from the 19-channel EEG dataset. The left panel illustrates the baseline, where at each frequency the mean was taken over all available windows. The right panel illustrates fluctuation over time, where at each frequency the standard deviation was taken over all available windows. No between-group difference was found at any frequency, either in the mean or the fluctuation of spectral power. HC: healthy control; SZ: schizophrenia.


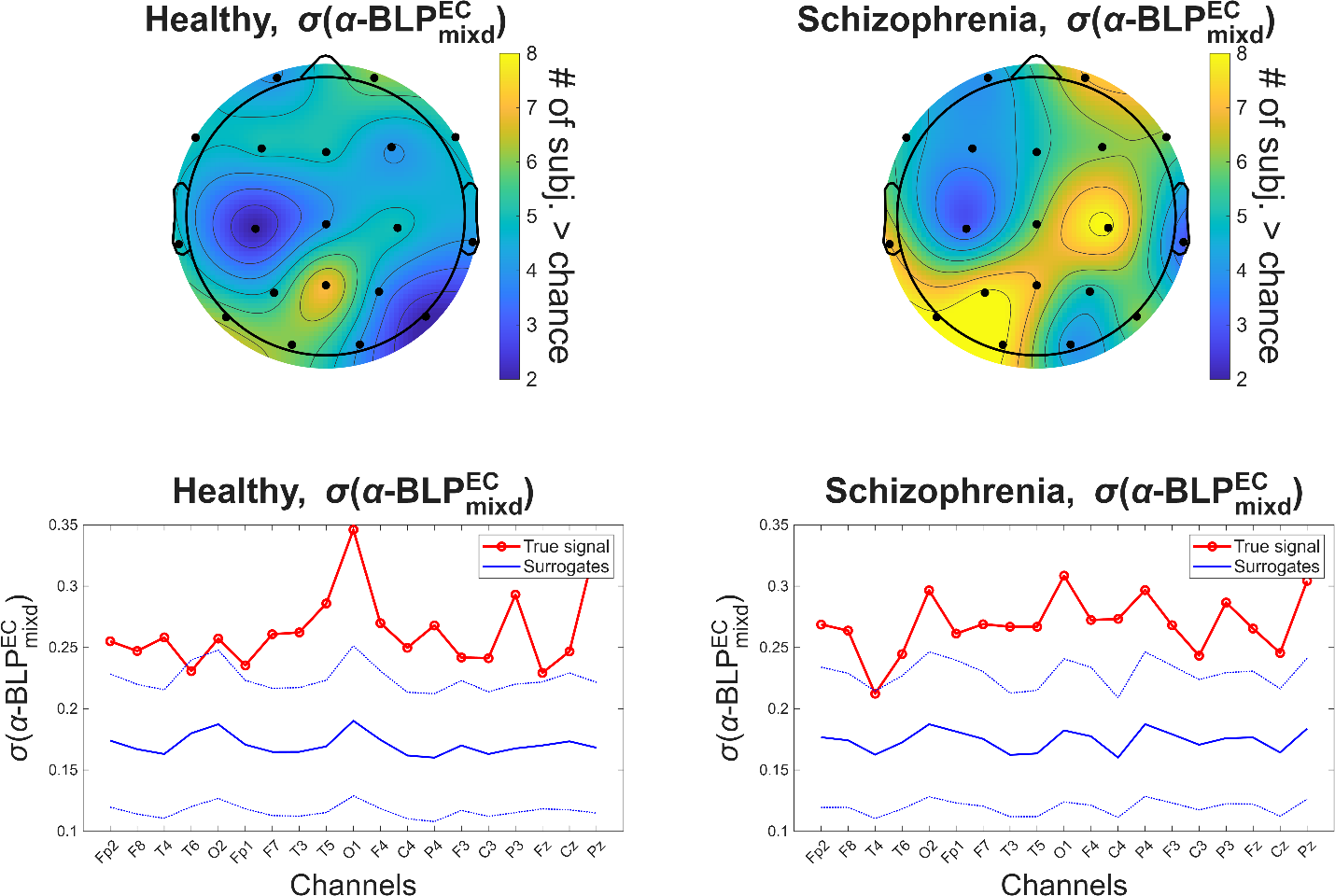


**Supplementary Figure S10.** Results of surrogate data analysis of alpha-band (8-13 Hz) activity in HC (left) and SZ (right) groups on the 19-channel EEG dataset. Upper panels: number of participants for given locations where nonlinearity could be confirmed. Lower panels: grand average true $\sigma(\alpha{BLP}_{mixd}^{EC})$ (red) contrasted with those obtained from surrogate data (blue). Continuous line denotes the mean, while dotted line denotes standard deviation from the mean.


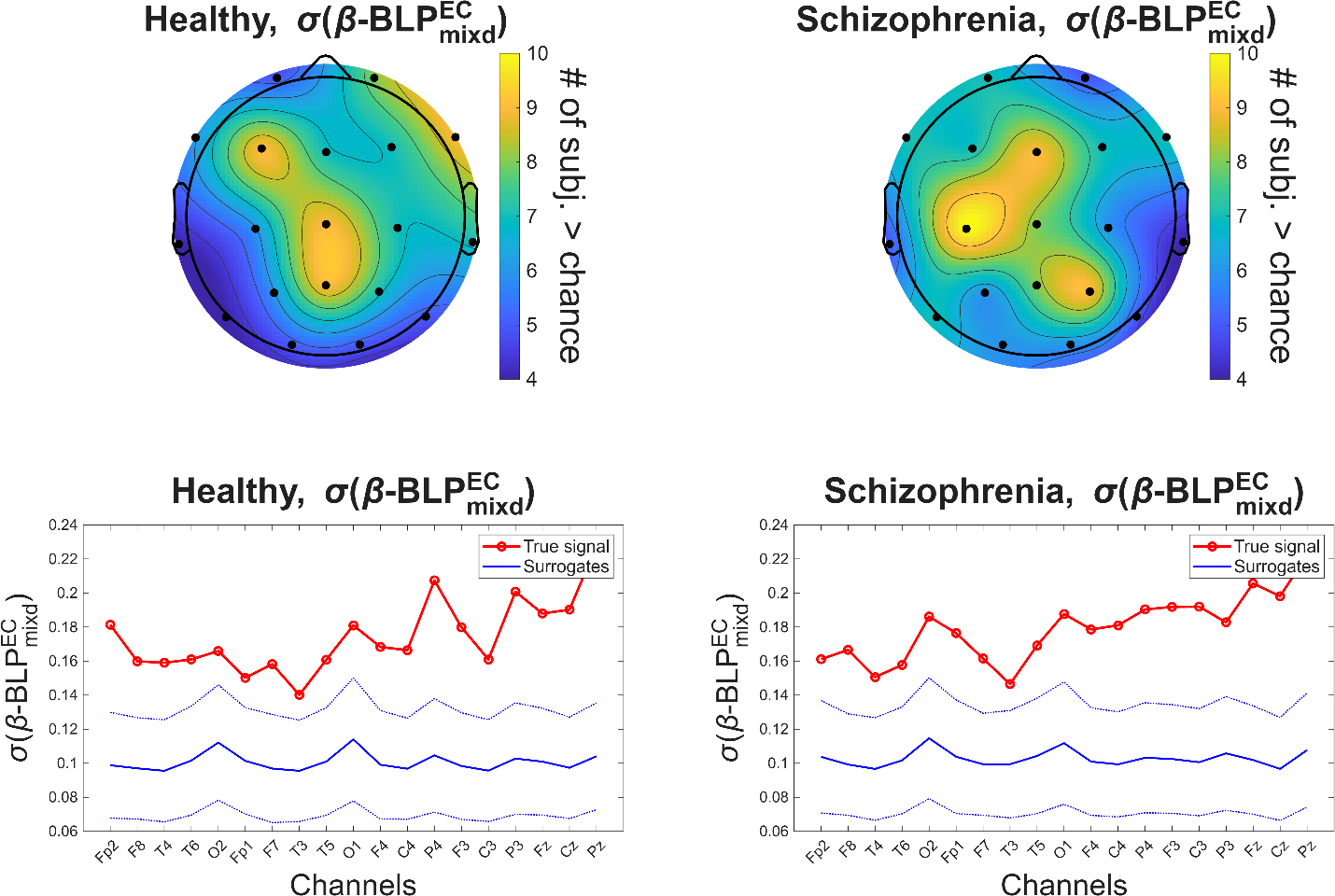


**Supplementary Figure S11.** Results of surrogate data analysis of beta-band (13-25 Hz) activity in HC (left) and SZ (right) groups on the 19-channel EEG dataset. Upper panels: number of participants for given locations where nonlinearity could be confirmed. Lower panels: grand average true $\sigma(\alpha{BLP}_{mixd}^{EC})$ (red) contrasted with those obtained from surrogate data (blue). Continuous line denotes the mean, while dotted line denotes standard deviation from the mean.

# References

1. American Psychiatric Association, D. and A.P. Association, *Diagnostic and statistical manual of mental disorders: DSM-5*. Vol. 5. 2013: American psychiatric association Washington, DC.

2. Kay, S.R., A. Fiszbein, and L.A. Opler, *The positive and negative syndrome scale (PANSS) for schizophrenia.* Schizophr Bull, 1987. **13**(2): p. 261-76.

3. Olejarczyk, E. and W. Jernajczyk, *Graph-based analysis of brain connectivity in schizophrenia.* PLoS One, 2017. **12**(11): p. e0188629.
